# Poor clinical outcomes associated with suboptimal antibiotic treatment among older long-term care facility residents with urinary tract infection: a retrospective cohort study

**DOI:** 10.1186/s12877-021-02378-5

**Published:** 2021-07-23

**Authors:** Haley J. Appaneal, Theresa I. Shireman, Vrishali V. Lopes, Vincent Mor, David M. Dosa, Kerry L. LaPlante, Aisling R. Caffrey

**Affiliations:** 1 Infectious Diseases Research Program, Providence Veterans Affairs Medical Center, 830 Chalkstone Ave, Providence, RI 02908 USA; 2 Center of Innovation in Long-Term Support Services, Providence Veterans Affairs Medical Center, Providence, RI USA; 3 College of Pharmacy, University of Rhode Island, Kingston, RI USA; 4 Center for Gerontology & Health Care Research and Department of Health Services Policy & Practice, Brown University School of Public Health, Providence, RI USA; 5 Division of Infectious Diseases, Warren Alpert Medical School of Brown University, Providence, RI USA

**Keywords:** Urinary tract infection, Suboptimal antibiotic treatment, Veterans affairs, Community living center

## Abstract

**Background:**

Antibiotic use is associated with several antibiotic-related harms in vulnerable, older long-term care (LTC) residents. Suboptimal antibiotic use may also be associated with harms but has not yet been investigated. The aim of this work was to compare rates of poor clinical outcomes among LTC residents with UTI receiving suboptimal versus optimal antibiotic treatment.

**Methods:**

We conducted a retrospective cohort study among residents with an incident urinary tract infection (UTI) treated in Veterans Affairs LTC units (2013–2018). Potentially suboptimal antibiotic treatment was defined as use of a suboptimal initial antibiotic drug choice, dose frequency, and/or excessive treatment duration. The primary outcome was time to a composite measure of poor clinical outcome, defined as UTI recurrence, acute care hospitalization/emergency department visit, adverse drug event, *Clostridioides difficile* infection (CDI), or death within 30 days of antibiotic discontinuation. Shared frailty Cox proportional hazard regression models were used to compare the time-to-event between suboptimal and optimal treatment.

**Results:**

Among 19,701 LTC residents with an incident UTI, 64.6% received potentially suboptimal antibiotic treatment and 35.4% experienced a poor clinical outcome. In adjusted analyses, potentially suboptimal antibiotic treatment was associated with a small increased hazard of poor clinical outcome (aHR 1.06, 95% CI 1.01–1.11) as compared with optimal treatment, driven by an increased hazard of CDI (aHR 1.94, 95% CI 1.54–2.44).

**Conclusion:**

In this national cohort study, suboptimal antibiotic treatment was associated with a 6% increased risk of the composite measure of poor clinical outcomes, in particular, a 94% increased risk of CDI. Beyond the decision to use antibiotics, clinicians should also consider the potential harms of suboptimal treatment choices with regards to drug type, dose frequency, and duration used.

**Supplementary Information:**

The online version contains supplementary material available at 10.1186/s12877-021-02378-5.

## Background

Antibiotic resistance poses one of the most urgent and widespread threats to public health and is driven by antibiotic use [1, 2]. Antibiotics use is highly prevalent in long-term care facilities (LTCFs) [3]. Antibiotics are used frequently in older residents for many reasons, including higher risk of infection due to immunosuppression, malnutrition, dehydration, presence of multiple comorbidities, and functional impairment [4–7]. Several other issues such as diagnostic uncertainty, the atypical and/or subtle presentation of common infections, and the frequency of colonization with antibiotic-resistant pathogens also contribute to antibiotic use in this population [5]. Approximately 10% of residents are on at least one antibiotic at any one time and between 50 and 75% of residents receive an antibiotic over the course of a year [8–11]. Up to 75% of antibiotic treatment in LTCFs may be inappropriate, putting already vulnerable residents at risk for unintended consequences of antibiotic use, including selection for colonization or infection with resistant organisms and *Clostridioides difficile*, antibiotic allergies, and other adverse drug effects and drug toxicities [12–14].

The most common driver of antibiotic use and reason for inappropriate antibiotic use in LTCFs is urinary tract infection (UTI) [15]. A study which included one-day point prevalence surveys from nine nursing homes in four states demonstrated that one in three antibiotics given to residents are for UTI and at least half of these antibiotics are inappropriate with regards to the drug choice, dose, or duration given [16]. Other studies have estimated that between 25 and 85% of antibiotic prescriptions for residents with UTIs are inappropriate [17–20].

Prior studies have assessed the harms associated with unnecessary antibiotic use [18, 21, 22]. No previous studies have assessed the harms associated with suboptimal antibiotic use, whether or not the antibiotics were necessary or unnecessary. Suboptimal antibiotic treatment as assessed by drug choice, dose, and/or duration, in already frail, older residents could also be associated with other poor clinical outcomes such as infection recurrence, acute care hospitalizations, visits to the emergency department, or even mortality [23, 24]. The aims of this study were to compare rates of poor clinical outcomes among LTCF residents with UTI receiving suboptimal and optimal antibiotic treatment. Our study focused on suboptimal antibiotic treatment, which has previously been defined as use of antibiotics in the setting of established infection that can be improved [25]. In clinical practice, the antibiotic-decision making process is complex and many factors need to be considered to ensure that optimal therapy is used [12, 26]. Therefore, we sought to assess the clinical impact of several domains of suboptimal antibiotic treatment, including the drug choice, dose frequency, and/or duration of therapy, all of which have the potential to be improved should a negative clinical impact be observed.

## Methods

The study design and methods were defined a priori in the study protocol, which was approved by the Institutional Review Board (IRB) and the Research and Development (R&D) Committee of the Providence Veterans Affairs Medical Center prior to initiation with a waiver of the informed consent process. All methods were carried out in accordance with relevant guidelines and regulations.

### Data sources, study design, setting, and population

**Fig. 1:**
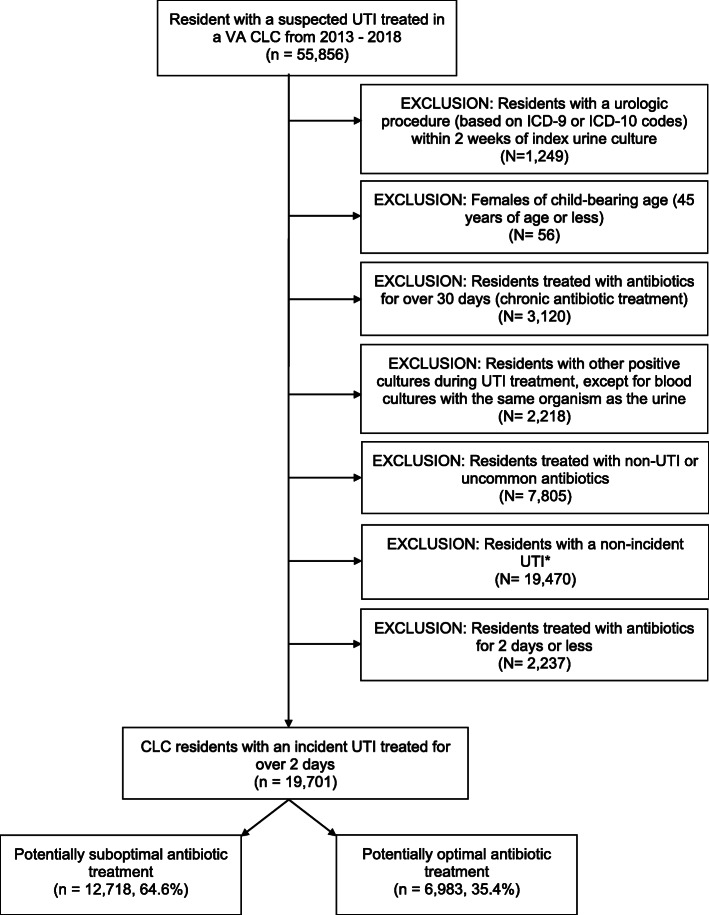
Flow chart for study population. CLC = Community Living Center, ICD-9/ICD-10 = International Classification of Diseases, 9th or 10th Revision diagnosis codes, UTI = Urinary tract infection; VA = Veterans Affairs. Figure adapted from previously published work [27].

We used data from the national VA Corporate Data Warehouse including microbiology reports, inpatient and outpatient care, diagnoses, procedures, surgeries, demographics, vital status, inpatient and outpatient medications, laboratory, and vital measurements. We conducted a retrospective cohort study of residents with a suspected incident UTI (first during the study with none in the year prior) treated in VA long-term care units (known as community living centers or CLCs) from January 2013 to December 2018. We included residents with incident CLC UTIs, however they could have had a UTI in another setting (e.g. hospital or outpatient) previously. Figure 1 presents a flow chart of methods used to identify our study population [27, 28]. Suspected UTIs required the collection of a urine culture (despite whether or not the culture was ultimately positive or negative for microbial growth) and an antibiotic given on the culture collection date or within 3 days after culture collection [27, 28]. We excluded residents with a recent urologic procedure, female residents of childbearing age, those treated with antibiotics for over 30 days, those with other positive cultures, and those treated with any non-UTI or uncommon antibiotics. We also excluded residents who were treated with antibiotics for 2 days or less.

### Exposure

We compared residents with UTIs receiving potentially suboptimal antibiotic treatment with those receiving potentially optimal antibiotic treatment. Potentially suboptimal antibiotic treatment was defined as use of a suboptimal initial antibiotic drug choice, dose frequency, and/or excessive treatment duration [27]. Initial drug choice was considered suboptimal based on resistance to the antibiotics used on day 1 of treatment, considering the resident’s urine culture and susceptibility results in the previous 180 days. If no urine culture and susceptibility data were available, the initial agents used were considered suboptimal if they provided insufficient coverage (susceptibility < 80% based on the CLC urine antibiogram for the year prior). Dose frequency was considered suboptimal based on renal function. In the case of missing serum creatine, dose frequency was defined as suboptimal, as clinicians would be unable to perform renal dose adjustment without an estimate of creatine clearance. Antibiotic duration was considered excessive if was ≥ 14 days.

### Outcomes

The primary outcome of interest was time to a composite measure of any of the following poor clinical outcomes within 30 days of antibiotic discontinuation: UTI recurrence, acute care hospitalization or emergency department (ED) visit, adverse drug event, *Clostridioides difficile* infection (CDI), or death. For residents who experienced more than one of these poor clinical outcomes of interest we considered the event that occurred first. For secondary outcomes, we assessed time to each poor clinical outcome separately. We censored residents who were alive and without the event of interest after 30 days. Table 1 presents study definitions used for primary and secondary outcomes.

**Table 1:** Study definitions used to measure composite and individual poor clinical outcomes

Outcome	Definition	Methods
**Primary outcome (composite outcome)**		
Poor clinical outcome	UTI recurrence, acute care hospitalization or ED visit, adverse drug event, CDI, or death	Each individual clinical outcome defined as below.
**Secondary outcomes (individual poor clinical outcomes)**		
UTI recurrence	Subsequent UTI after the first incident UTI	UTI recurrences were identified using the same methods to identify the first incident UTI. Required urine culture collection and an antibiotic given on the culture collection date or within 3 days after culture collection.
Acute care hospitalization or ED visit	Any admission to an acute care unit or ED within 30 days of end of antibiotic treatment, except for those for an elective hip or knee procedure	VA hospitalizations were captured using VA inpatient admission data. VA ED visits were captured using VA outpatient visit data. Non-VA hospitalizations and ED visits VA Fee basis files were captured using VA Fee basis files. ICD-9 or 10 procedure codes were used to identify elective hip or knee procedures (see supplement for codes).
Antibiotic related adverse drug events	Diagnosis of an antibiotic related adverse drug event	Previously published ICD-9 or 10 lists were used to identify antibiotic related adverse drug events (see supplement for codes) [29, 30].
CDI	Laboratory evidence of CDI or diagnosis of CDI	Positive stool sample for *C. difficile* toxin(s) or ICD-9 or 10 diagnosis code for CDI (008.45, A04.71, A04.72) [31, 32].
All-cause mortality	Death due to any cause	The Vital Status Mini File was used to confirm date of death.

*CDI Clostridioides difficile* infection, *ED* Emergency Department, *ICD-9 or 10* International Classification of Diseases, Ninth and Tenth Edition, Clinical Modification, *UTI* Urinary tract infection

### Analytic approach

We compared the composite measure of poor clinical outcomes, as well as the individual clinical outcomes, among residents with incident UTIs receiving potentially suboptimal and optimal antibiotic treatment. Differences between groups were analyzed using chi-square for categorical data and Student’s *t* test or the Wilcoxon rank sum test for continuous data, as appropriate. To account for the multi-level structure of our data (individual residents with UTIs clustered within CLCs), CLC was included a random effect in our models with log-normal distribution to represent shared frailty among residents of the same CLC [33]. Cox models with mixed effects were used to compare the primary (time to composite poor clinical outcome) and secondary outcomes (time to each individual clinical outcome) between groups and to estimate crude and adjusted hazard ratios (aHRs) and 95% confidence intervals (CI) [33]. The adjusted multivariable models included resident-level predictors of suboptimal antibiotic treatment and that were also associated with the poor clinical outcomes [27]. We also adjusted for CLC-level variables predictive of suboptimal antibiotic treatment [34]. All predictors included in the adjusted analyses are presented in the results tables. Evaluation of assumptions and plotting of relevant model parameters were conducted, including for the assessment of proportionality [35].

We conducted subgroup analyses among residents who were initially treated with only one antibiotic class (fluroquinolones, cephalosporins, genitourinary tact agents, or other beta lactams). We also conducted subgroup analyses among residents who received only one subtype of suboptimal treatment (drug choice, dose frequency, or excessive treatment duration). Finally, we conducted sensitivity analyses with varying follow-up times (14, 60, 90, 180 days). We used a shorter 14-day follow-up time as some events such as UTI recurrence, acute care hospitalization or emergency department visits, and adverse drug events, tend to occur during or shortly after exposure to antibiotics [21, 36]. Other events, such as CDI, can occur several weeks to months after discontinuing antibiotic therapy. We used longer follow-up times (up 60, 90, and 180 days) to capture events that might develop beyond 30 days after antibiotic discontinuation (available in supplemental material) [37]. We used SAS (version 9.2) and R-studio (version 3.6.1) for our analyses.

## Results

From 2013 to 2018, we identified 19,701 CLC residents with an incident UTI treated in 120 VA CLCs treated for at least 3 days. Overall, 64.6% of residents (*n* = 12,718) received potentially suboptimal antibiotic treatment and 35.4% (*n* = 6983) received potentially optimal antibiotic treatment. The CLC at the median used potentially suboptimal antibiotic treatment in 66.3% of residents with an incident UTI (range from 36.8% for the CLC with the lowest suboptimal use to 100% for the CLC with the highest suboptimal use).

As expected, there were several differences in the baseline characteristics and initial (empiric) antibiotic treatment of residents receiving potentially suboptimal versus optimal antibiotic treatment (Table 2). Those receiving potentially optimal antibiotic treatment were more likely to have chronic renal disease (29.7% vs. 27.3%, *p* < 0.001) and exposure to any antibiotic in the last 30 days (44.4% vs. 42.0%), and less likely to have a UTI diagnosis in the year prior (34.3% vs. 40.3%), compared with those receiving optimal antibiotic treatment.

**Table 2:** Baseline characteristics, initial antibiotics and outcomes among residents with urinary tract infections receiving potentially suboptimal and optimal antibiotic treatment

	Potentially suboptimal antibiotic treatment (***n*** = 12,718)	Potentially optimal antibiotic treatment (***n*** = 6983)	***P*** value
**Baseline Characteristics**			
Age in years, median (IQR)	75 (67–85)	74 (67–84)	p < 0.001
Male gender	12,208 (96)	6698 (95.9)	0.808
White race	9477 (74.5)	5266 (75.4)	0.166
Chronic renal disease comorbidity	3783 (29.7)	1905 (27.3)	< 0.001
Cardiopulmonary disease comorbidity	9156 (72)	5158 (73.9)	0.005
Genitourinary disease comorbidity	6733 (52.9)	3833 (54.9)	0.009
Urinary tract infection diagnosis in the year prior	4357 (34.3)	2815 (40.3)	< 0.001
Skin or soft tissue infection diagnosis in the year prior	2631 (20.7)	1266 (18.1)	< 0.001
Hospitalization, 30 days prior treatment	3708 (29.2)	2127 (30.5)	0.06
Exposure to any antibiotic, 30 days prior to treatment	5650 (44.4)	2932 (42.0)	0.001
Exposure to fluoroquinolone, 30 days prior to treatment	1840 (14.5)	776 (11.1)	< 0.001
**Initial Antibiotic Treatment**			
Fluoroquinolone	5403 (42.5)	1874 (26.8)	< 0.001
Ciprofloxacin	3972 (31.2)	1585 (22.7)	< 0.001
Levofloxacin	1458 (11.5)	292 (4.2)	< 0.001
Cephalosporin	3160 (24.8)	2274 (32.6)	< 0.001
Cephalexin	1076 (8.5)	287 (4.1)	< 0.001
Ceftriaxone	656 (5.2)	972 (13.9)	< 0.001
Typical genitourinary tract agent	2848 (22.4)	1356 (19.4)	< 0.001
Sulfamethoxazole/trimethoprim	2330 (18.3)	1070 (15.3)	< 0.001
Nitrofurantoin	510 (4)	273 (3.9)	0.366
Other beta lactam	2394 (18.8)	1575 (22.6)	< 0.001
Amoxicillin/clavulanate	1101 (8.7)	425 (6.1)	< 0.001
Piperacillin/tazobactam	687 (5.4)	579 (8.3)	< 0.001
Amoxicillin	487 (3.8)	328 (4.7)	0.003
Other antibiotics	1134 (8.9)	821 (11.8)	< 0.001
Vancomycin	975 (7.7)	711 (10.2)	< 0.001
Clindamycin	33 (0.3)	< 5	0.001
Treatment duration in days, median (interquartile range)	9 (7–12)	8 (6–10)	p < 0.001
**Outcomes**^a^			
Poor clinical outcome	4780 (37.6)	2557 (36.6)	0.179
Hospitalization/ Emergency department visit	2499 (19.6)	1386 (19.8)	0.737
UTI recurrence	1589 (12.5)	927 (13.3)	0.116
All-cause mortality	1418 (11.1)	703 (10.1)	0.019
*Clostridioides difficile* infection	339 (2.7)	98 (1.4)	< 0.001
Antibiotic related adverse drug event	27 (0.2)	17 (0.2)	0.658

*CLC* Community Living Center, *UTI* Urinary tract infection

Data are presented as n (%), unless otherwise specified

^a^Crude outcome rates at 30 days

We compared baseline characteristics, initial antibiotic treatment (day 1, empiric treatment) and outcomes among CLC residents with an incident UTI receiving potentially suboptimal antibiotic treatment and those receiving potentially optimal antibiotic treatment. We present a few significant differences in baseline characteristics in the Table above. Our previous work compares baseline characteristics (including socio-demographics, comorbidities, prior healthcare exposures, prior infections, prior antibiotic exposures, prior culture collection, and prior laboratory results) between groups in more detail [27].

Potentially suboptimal antibiotic treatment was defined as exposure to any subtype of potentially suboptimal antibiotic treatment: drug choice (based on previous urine cultures and susceptibilities or local CLC urine antibiogram), dose frequency (based on renal function), and/or longer than recommended duration (greater than 14 days)

The following agents were included in each class: fluoroquinolones (ciprofloxacin, levofloxacin), typical genitourinary tract agents (nitrofurantoin, sulfamethoxazole/ trimethoprim, fosfomycin, trimethoprim), cephalosporins (cefaclor, cefazolin, cefotetan, cefoxitin, cefuroxime, cephalexin, cefdinir, cefepime, cefixime, cefotaxime, cefpodoxime, ceftazidime, ceftriaxone), and other beta-lactams (amoxicillin, ampicillin, amoxicillin/clavulanate, amipicillin/sulbactam, imipenem, meropenem, ertapenem, doripenem, piperacillin/ tazobactam)

Overall, 37.2% (*n* = 7337) of residents with an incident UTI experienced a poor clinical outcome within 30 days of antibiotic discontinuation (Table 2). Time to event analyses comparing residents who received potentially suboptimal antibiotic treatment to those who received optimal antibiotic treatment are presented in Table 3. The median time to the first of any of the composite poor clinical outcomes was 10 days (IQR 4–18.5) in residents receiving potentially suboptimal antibiotic treatment and 11 days (IQR 5–19) in those receiving optimal antibiotic treatment, *p* = 0.001. In adjusted analyses, receipt of potentially suboptimal antibiotic treatment was associated with a small increased hazard of the composite poor clinical outcome (aHR 1.06, 95% CI 1.01–1.11) as compared with optimal antibiotic treatment. Receipt of potentially suboptimal antibiotic treatment was associated with an increased hazard of CDI (aHR 1.94, 95% CI 1.54–2.44), but not with any other individual clinical outcome assessed.

**Table 3:** Time to event analyses among CLC residents with UTI receiving potentially suboptimal and optimal antibiotic treatment

	Median time in days to event for residents who received potentially suboptimal treatment (***n*** = 12,718)	Median time in days to event for residents who received potentially optimal treatment (***n*** = 6983)	Unadjusted HR (95% CI)	Adjusted HR (95% CI)
Poor clinical outcome^a^	10 (4–18.5)	11 (5–19)	1.05 (1.00–1.10)	1.06 (1.01–1.11)
Hospitalization/ Emergency department visit^b^	11 (4–20)	12 (5–20)	0.99 (0.93–1.06)	1.02 (0.96–1.10)
UTI recurrence^c^	13 (7–21)	14 (8–21)	0.93 (0.86–1.01)	0.97 (0.89–1.05)
All-cause mortality^d^	9 (3–18)	9 (3–18)	1.11 (1.02–1.22)	1.05 (0.95–1.15)
*Clostridioides difficile* infection^e^	10 (3–17)	11 (5–17)	2.03 (1.62–2.54)	1.94 (1.54–2.44)
Antibiotic related adverse drug event^f^	6 (1–15)	11 (3–18)	0.89 (0.48–1.64)	0.93 (0.51–1.72)

*CI* Confidence interval, *CLC* Community Living Center, *HR* Hazard ratio, *UTI* Urinary tract infection, *VAMC* Veterans Affairs Medical Center

All covariates were included as dichotomous variables representing presence as compared to absence of the characteristic of interest, unless otherwise noted. Age was included as a categorical variable (≥ 85, 75–84, 65–74 years) as compared to < 65 years. Recent high WBC was included as a WBC > 10 × 10^3^/μL within 7 days prior to treatment as compared to a measurement below or missing. Total CLC incident UTI rate per 10,000 bed days was included as a continuous variable. Year of episode was included as a discrete variable for each year

^a^Adjusted for 8 resident-level covariates (genitourinary disease comorbidity, cardiopulmonary comorbidity, chronic renal disease comorbidity, previous fluroquinolone exposure in the past 30 days, age, previous VAMC hospitalization in the past 30 days, previous VAMC urine culture in the past 365 days, high white blood cell count), and 1 CLC-level covariate (total CLC incident UTI rate per 10,000 bed days)

^b^Adjusted for 10 resident-level covariates (cardiopulmonary comorbidity, genitourinary disease comorbidity, chronic renal disease comorbidity, age, previous VAMC hospitalization in the past 30 days, previous outpatient VA urine culture in the past 365 days, previous CLC urine culture in the past 365 days, previous VAMC urine culture in the past 365 days, high white blood cell count, year of episode) and 1 CLC-level covariate (total CLC incident UTI rate per 10,000 bed days)

^c^Adjusted for 5 resident-level covariates (genitourinary disease comorbidity, previous fluroquinolone exposure in the past 30 days, previous fluroquinolone resistant culture in the past 365 days, previous CLC urine culture in the past 365 days, previous VAMC urine culture in the past 365 days), and 1 CLC-level covariate (total CLC incident UTI rate per 10,000 bed days)

^d^Adjusted for 10 resident-level covariates (history of a skin infection diagnosis in the past 365 days, history of a urinary tract infection diagnosis in the past 365 days, chronic renal disease comorbidity, age, previous fluroquinolone exposure in the past 30 days, previous VAMC hospitalization in the past 30 days, previous outpatient VA urine culture in the past 365 days, previous VAMC urine culture in the past 365 days, high white blood cell count, year of episode), and 1 CLC-level covariate (total CLC incident UTI rate per 10,000 bed days)

^e^Adjusted for 8 resident-level covariates (history of a skin infection diagnosis in the past 365 days, genitourinary disease comorbidity, chronic renal disease comorbidity, previous VAMC hospitalization in the past 30 days, previous CLC urine culture in the past 365 days, previous VAMC urine culture in the past 365 days, high white blood cell count, year of episode), and 1 CLC-level covariate (total CLC incident UTI rate per 10,000 bed days)

^f^Adjusted for 2 resident-level covariates (VAMC hospitalization in the past 30 days, year of episode), and 1 CLC-level covariate (total CLC incident UTI rate per 10,000 bed days)

All covariates were included in adjusted models as dichotomous variables representing presence as compared to absence of the characteristic of interest, unless otherwise noted. Age was included as a categorical variable (≥ 85, 75–84, 65–74 years) as compared to < 65 years. Recent high WBC was included as a WBC > 10 × 10^3^/μL within 7 days prior to treatment as compared to a measurement below or missing. Total CLC incident UTI rate per 10,000 bed days was included as a continuous variable. Year of episode was included as a discrete variable for each year

Results of subgroup analyses among those who initially received only one antibiotic class can be found in Table 4. Potentially suboptimal treatment was not associated with an increased hazard of the composite poor clinical outcome in any of the subgroups of residents who were initially treated with one class of antibiotics alone (fluroquinolones, cephalosporins, genitourinary tact agents, or other beta lactams). However, among those initially treated with fluoroquinolones alone (aHR 1.91, 95% CI1.09–3.36) and among those who initially received cephalosporins alone (aHR 1.93, 95% CI 1.12–3.31), potentially suboptimal treatment was associated with an increased hazard of CDI.

**Table 4:** Time to event analyses among CLC residents who initially received only one antibiotic class

Outcome	Initial Antibiotic Treatment	Potentially suboptimal treatment [n events/n total, (%)]	Potentially optimal treatment [n events/n total, (%)]	Unadjusted HR (95% CI)	Adjusted HR (95% CI)
Poor clinical outcome^a^	Fluroquinolone	1581/4460 (35.4%)	588/1672 (35.2%)	1.03 (0.94–1.14)	1.05 (0.95–1.16)
	Cephalosporin	722/1882 (38.4%)	661/1854 (35.7%)	1.07 (0.96–1.20)	1.06 (0.95–1.18)
	Genitourinary tract agent	809/2384 (33.9%)	415/1280 (32.4%)	1.06 (0.94–1.20)	1.09 (0.96–1.23)
	Other beta lactam	599/1578 (38.0%)	432/1087 (39.7%)	0.97 (0.85–1.09)	1.00 (0.88–1.13)
Hospitalization/ Emergency department visit^b^	Fluroquinolone	771/4460(17.3%)	312/1672 (18.7%)	0.95 (0.83–1.09)	1.01 (0.88–1.16)
	Cephalosporin	406/1882(21.6%)	346/1854(18.7%)	1.08 (0.93–1.26)	1.09 (0.94–1.27)
	Genitourinary tract agent	386/2384(16.2%)	217/1280(17.0%)	0.97 (0.82–1.14)	0.96 (0.81–1.15)
	Other beta lactam	346/1578(21.9%)	243/1087(22.4%)	1.01 (0.86–1.20)	1.11 (0.94–1.32)
UTI recurrence^c^	Fluroquinolone	522/4460(11.7%)	201/1672(12.0%)	0.97 (0.82–1.14)	1.00 (0.84–1.19)
	Cephalosporin	252/1882(13.4%)	251/1854(13.5%)	0.95 (0.80–1.14)	0.95 (0.79–1.14)
	Genitourinary tract agent	372/2384(15.6%)	169/1280(13.2%)	1.04 (0.86–1.25)	1.06 (0.87–1.30)
	Other beta lactam	180/1578(11.4%)	177/1087(16.3%)	0.69 (0.56–0.85)	0.78 (0.63–0.97)
All-cause mortality^d^	Fluroquinolone	527/4460(11.8%)	173/1672(10.3%)	1.16 (0.98–1.38)	1.07 (0.89–1.28)
	Cephalosporin	197/1882(10.5%)	182/1854(9.8%)	1.09 (0.88–1.33)	1.06 (0.86–1.30)
	Genitourinary tract agent	228/2384(9.6%)	93/1280(7.3%)	1.29 (1.01–1.64)	1.26 (0.98–1.63)
	Other beta lactam	187/1578(11.9%)	108/1087(9.9%)	1.19 (0.94–1.51)	1.15 (0.89–1.48)
*Clostridioides difficile* infection^e^	Fluroquinolone	66/4460(1.5%)	16/1672(1.0%)	1.56 (0.90–2.71)	1.91 (1.09–3.36)
	Cephalosporin	41/1882(2.2%)	20/1854(1.1%)	1.99 (1.16–3.43)	1.93 (1.12–3.31)
	Genitourinary tract agent	25/2384(1.0%)	12/1280(0.9%)	1.14 (0.57–2.28)	1.14 (0.55–2.35)
	Other beta lactam	24/1578(1.5%)	15/1087(1.4%)	1.10 (0.58–2.10)	1.35 (0.69–2.65)
Antibiotic related adverse drug event^f^	Fluroquinolone	< 0.5%	< 0.5%	0.87 (0.23–3.38)	1.02 (0.26–3.97)
	Cephalosporin	< 0.5%	< 0.5%	1.14 (0.28–4.68)	1.19 (0.29–4.95)
	Genitourinary tract agent	< 0.5%	< 0.5%	1.10 (0.20–6.03)	1.13 (0.21–6.18)
	Other beta lactam	< 0.5%	< 0.5%	1.15 (0.27–4.81)	1.14 (0.27–4.75)

*CI* Confidence interval, *CLC* Community Living Center, *HR* Hazard ratio, *UTI* Urinary tract infection, *VAMC* Veterans Affairs Medical Center

^a^Adjusted for 8 resident-level covariates (genitourinary disease comorbidity, cardiopulmonary comorbidity, chronic renal disease comorbidity, previous fluroquinolone exposure in the past 30 days, age, previous VAMC hospitalization in the past 30 days, previous VAMC urine culture in the past 365 days, high white blood cell count), and 1 CLC-level covariate (total CLC incident UTI rate per 10,000 bed days)

^b^Adjusted for 10 resident-level covariates (cardiopulmonary comorbidity, genitourinary disease comorbidity, chronic renal disease comorbidity, age, previous VAMC hospitalization in the past 30 days, previous outpatient VA urine culture in the past 365 days, previous CLC urine culture in the past 365 days, previous VAMC urine culture in the past 365 days, high white blood cell count, year of episode) and 1 CLC-level covariate (total CLC incident UTI rate per 10,000 bed days)

^c^Adjusted for 5 resident-level covariates (genitourinary disease comorbidity, previous fluroquinolone exposure in the past 30 days, previous fluroquinolone resistant culture in the past 365 days, previous CLC urine culture in the past 365 days, previous VAMC urine culture in the past 365 days), and 1 CLC-level covariate (total CLC incident UTI rate per 10,000 bed days)

^d^Adjusted for 10 resident-level covariates (history of a skin infection diagnosis in the past 365 days, history of a urinary tract infection diagnosis in the past 365 days, chronic renal disease comorbidity, age, previous fluroquinolone exposure in the past 30 days, previous VAMC hospitalization in the past 30 days, previous outpatient VA urine culture in the past 365 days, previous VAMC urine culture in the past 365 days, high white blood cell count, year of episode), and 1 CLC-level covariate (total CLC incident UTI rate per 10,000 bed days)

^e^Adjusted for 8 resident-level covariates (history of a skin infection diagnosis in the past 365 days, genitourinary disease comorbidity, chronic renal disease comorbidity, previous VAMC hospitalization in the past 30 days, previous CLC urine culture in the past 365 days, previous VAMC urine culture in the past 365 days, high white blood cell count, year of episode), and 1 CLC-level covariate (total CLC incident UTI rate per 10,000 bed days)

^f^Adjusted for 2 resident-level covariates (VAMC hospitalization in the past 30 days, year of episode), and 1 CLC-level covariate (total CLC incident UTI rate per 10,000 bed days)

All covariates were included in adjusted models as dichotomous variables representing presence as compared to absence of the characteristic of interest, unless otherwise noted. Age was included as a categorical variable (≥ 85, 75–84, 65–74 years) as compared to < 65 years. Recent high WBC was included as a WBC > 10 × 10^3^/μL within 7 days prior to treatment as compared to a measurement below or missing. Total CLC incident UTI rate per 10,000 bed days was included as a continuous variable. Year of episode was included as a discrete variable for each year

Results among those who received only one subtype of suboptimal treatment (suboptimal drug choice, dose frequency, or longer than recommended duration) as compared with optimal treatment can be found in Table 5. In adjusted analyses, receipt of suboptimal dose frequency was associated with an increased hazard of the composite poor clinical outcome (aHR 1.29, 95% CI 1.20–1.39). Suboptimal drug choice and longer than recommended treatment duration were not associated with the composite poor clinical outcome. Suboptimal dose frequency was associated with an increased hazard of hospitalization/ED visit (aHR 1.13, 95% CI 1.02–1.26), all-cause mortality (aHR 1.44, 95% CI 1.26–1.65), and CDI (aHR 3.21, 95% CI 2.42–4.25). Longer than recommended treatment duration was associated with a decreased hazard of mortality (aHR 0.71, 95% CI 0.55–0.90).

**Table 5:** Time to event-analyses for CLC residents received only one subtype of potentially suboptimal treatment (drug choice, dose frequency, or excessive treatment duration) as compared to optimal treatment

Outcome	Subtype of potentially suboptimal treatment	Unadjusted HR	Lower 95% CI	Upper 95% CI	Adjusted HR	Lower 95% CI	Upper 95% CI
Poor clinical outcome^a^	Suboptimal drug choice	0.91	0.86	0.97	0.96	0.91	1.03
	Suboptimal dose frequency	1.35	1.25	1.45	1.29	1.20	1.39
	Longer than recommended treatment duration	0.92	0.83	1.03	0.90	0.81	1.01
Hospitalization/ Emergency department visit^b^	Suboptimal drug choice	0.87	0.80	0.94	0.95	0.87	1.04
	Suboptimal dose frequency	1.19	1.07	1.31	1.13	1.02	1.26
	Longer than recommended treatment duration	1.08	0.94	1.25	1.03	0.89	1.18
UTI recurrence^c^	Suboptimal drug choice	0.86	0.78	0.95	0.93	0.84	1.04
	Suboptimal dose frequency	1.14	1.01	1.30	1.13	0.99	1.28
	Longer than recommended treatment duration	0.88	0.73	1.06	0.85	0.70	1.02
All-cause mortality^d^	Suboptimal drug choice	1.00	0.89	1.12	0.93	0.82	1.04
	Suboptimal dose frequency	1.52	1.33	1.73	1.44	1.26	1.65
	Longer than recommended treatment duration	0.68	0.53	0.86	0.71	0.55	0.90
*Clostridioides difficile* infection^e^	Suboptimal drug choice	0.92	0.67	1.25	1.04	0.75	1.45
	Suboptimal dose frequency	3.51	2.66	4.63	3.21	2.42	4.25
	Longer than recommended treatment duration	1.64	1.05	2.58	1.37	0.87	2.15
Antibiotic related adverse drug event^f^	Suboptimal drug choice	0.58	0.25	1.35	0.68	0.29	1.59
	Suboptimal dose frequency	1.50	0.65	3.48	1.38	0.59	3.20
	Longer than recommended treatment duration	1.21	0.35	4.12	1.20	0.35	4.11

*CI* Confidence interval, *CLC* Community Living Center, *HR* Hazard ratio, *UTI* Urinary tract infection, *VAMC* Veterans Affairs Medical Center

^a^Adjusted for 8 resident-level covariates (genitourinary disease comorbidity, cardiopulmonary comorbidity, chronic renal disease comorbidity, previous fluroquinolone exposure in the past 30 days, age, previous VAMC hospitalization in the past 30 days, previous VAMC urine culture in the past 365 days, high white blood cell count), and 1 CLC-level covariate (total CLC incident UTI rate per 10,000 bed days)

^b^Adjusted for 10 resident-level covariates (cardiopulmonary comorbidity, genitourinary disease comorbidity, chronic renal disease comorbidity, age, previous VAMC hospitalization in the past 30 days, previous outpatient VA urine culture in the past 365 days, previous CLC urine culture in the past 365 days, previous VAMC urine culture in the past 365 days, high white blood cell count, year of episode) and 1 CLC-level covariate (total CLC incident UTI rate per 10,000 bed days)

^c^Adjusted for 5 resident-level covariates (genitourinary disease comorbidity, previous fluroquinolone exposure in the past 30 days, previous fluroquinolone resistant culture in the past 365 days, previous CLC urine culture in the past 365 days, previous VAMC urine culture in the past 365 days), and 1 CLC-level covariate (total CLC incident UTI rate per 10,000 bed days)

^d^Adjusted for 10 resident-level covariates (history of a skin infection diagnosis in the past 365 days, history of a urinary tract infection diagnosis in the past 365 days, chronic renal disease comorbidity, age, previous fluroquinolone exposure in the past 30 days, previous VAMC hospitalization in the past 30 days, previous outpatient VA urine culture in the past 365 days, previous VAMC urine culture in the past 365 days, high white blood cell count, year of episode), and 1 CLC-level covariate (total CLC incident UTI rate per 10,000 bed days)

^e^ Adjusted for 8 resident-level covariates (history of a skin infection diagnosis in the past 365 days, genitourinary disease comorbidity, chronic renal disease comorbidity, previous VAMC hospitalization in the past 30 days, previous CLC urine culture in the past 365 days, previous VAMC urine culture in the past 365 days, high white blood cell count, year of episode), and 1 CLC-level covariate (total CLC incident UTI rate per 10,000 bed days)

^f^Adjusted for 2 resident-level covariates (VAMC hospitalization in the past 30 days, year of episode), and 1 CLC-level covariate (total CLC incident UTI rate per 10,000 bed days)

In sensitivity analyses, potentially suboptimal antibiotic treatment was associated with an increased hazard of the composite poor clinical outcome using a shorter follow-up of 14 days, but was not with a longer follow-up of 60, 90, or 180-days (see Supplementary Table 1). Potentially suboptimal antibiotic treatment was not associated with an alternate definition of the composite poor clinical outcome which excluded CDI, except in the shortest follow-up period of 14 days (see Supplementary Table 2*).*

## Discussion

To our knowledge, this study is the first to focus specifically on comparing rates of poor clinical outcomes among residents with UTI based on suboptimal versus optimal antibiotic treatment. This work suggests an association between suboptimal antibiotic treatment and poor clinical outcomes driven by a 94% increased risk of CDI. Our study is the first to identify an increased risk of CDI among residents with UTI who did not receive an optimal antibiotic, and/or dose frequency, and/or duration, despite whether the treatment was necessary or unnecessary.

Our work advances previous work indicating antibiotic use is associated with a number of unintended harms in LTCF residents [18, 38, 39]. Among 110,656 older adults residing in 607 nursing homes in Canada, residence alone in nursing homes with high antibiotic use was associated with an increased risk of residents experiencing an antibiotic-related adverse event (adjusted odds ratio, 1.24; 95% CI, 1.07–1.42; *p* = 0.003) [39]. Work in other settings has similarly confirmed that antibiotic-related harms are common. In the hospital setting, it is estimated that about 20% of admitted patients treated with antibiotics develop antibiotic-associated adverse events, including CDI, development of resistant infections, and other gastrointestinal, cardiac, renal, hepatobiliary, neurologic, dermatologic, and musculoskeletal adverse manifestations [21, 40, 41]. In the outpatient setting, it is well established that antibiotics are among the most common cause of adverse drug events [42–44]. Antibiotics are associated with up to 20% of all ED visits for drug-related adverse events among adults in the United States [41, 43]. In our study, 37% of residents treated with antibiotics for incident UTI experienced a poor clinical outcome.

Our work also corresponds with existing research on unnecessary antibiotic treatment and associated harms, including CDI, acquisition of drug-resistant pathogens, and other adverse events [22, 45, 46]. In a retrospective study of 172 residents with suspected UTI from two Rhode Island nursing homes, residents who received antibiotics but did not meet criteria for treatment were 8.5 times (95% CI, 1.7–42.2) more likely to develop CDI than the rest of the nursing home population [18]. This is also observed in the hospital setting [22, 47]. While these previous studies demonstrate an association between unnecessary antibiotic use and the development of avoidable adverse events, our work advances these findings, demonstrating an association between suboptimal antibiotic treatment and the development of poor outcomes. In contrast to previous studies which have assessed unnecessary treatment, our study assessed residents who did not receive the preferred antibiotic, and/or dose frequency, and/or duration regardless of treatment necessity.

Our results were largely driven by an increased risk of CDI associated with suboptimal antibiotic treatment among residents with UTI. The risk of CDI was 1.94 times higher among residents who received potentially suboptimal antibiotic treatment as compared to those who received optimal antibiotic treatment. It is well-known that antibiotic use is one of the most important risk factors for CDI, and that any antibiotic exposure can lead to disruption of the normal gastrointestinal microbiota and result in CDI. Prior work among nursing home residents, demonstrated that a 7-day antibiotic course, compared with no antibiotic treatment, was associated with a 1.8 times increased risk of CDI [48]. This prior work also demonstrated that the risk of CDI varies by antibiotic agent/class and duration of treatment [48]. Previous work has demonstrated broad-spectrum antibiotics, such as clindamycin, fluoroquinolones, cephalosporins, and amoxicillin/clavulanate, are associated with a higher risk of CDI than other antibiotics [48–51]. Longer durations of treatment have also been shown to increase the risk of CDI, potentially through increased perturbation of the colonic microbiota [52, 53]. Our results are thus likely related to differences in treatment between residents receiving potentially suboptimal antibiotic treatment and optimal antibiotic treatment, which is expected as our definition of suboptimal treatment was based on drug choice, dose frequency, and duration of treatment. For example, residents who received suboptimal antibiotics were more likely to be treated with higher risk antibiotics such as fluoroquinolones, some cephalosporins, amoxicillin/clavulanate, and clindamycin than those who received optimal antibiotic treatment. In subgroup analyses, potentially suboptimal antibiotic treatment was associated with an increased hazard of CDI in those who initially received monotherapy with antibiotics known to increase the risk of CDI, including those initially treated with fluoroquinolones alone and those treated with cephalosporins alone. Fluoroquinolones may have been more likely to be suboptimal due to high rates of resistance among urinary isolates in the VA [54].

Treatment duration was longer among those who received suboptimal antibiotic treatment. A recent longitudinal case-cohort study among residents of nursing homes in Ontario, Canada found that antibiotic choice and duration had a significant impact on risk for CDI [48]. Compared to a 7 day course of antibiotics, a 10-day course was associated with a 12% higher risk for CDI (adjusted relative risk [aRR] 1.12, 95% CI 1.09–1.14) and a 14 day course was associated with a 27% higher risk (aRR 1.12, 95% CI 1.21–1.30) [48]. Authors of previous work did not define whether courses were necessary/unnecessary or optimal/suboptimal, but rather just compared the risk of CDI for any 10- or 14-day course of antibiotics to any 7-day course. In previous work when comparing risk of CDI for agents used for 7 days, fluoroquinolone, clindamycin, and cephalosporin agents demonstrated the highest risk for CDI development [48]. However, in our study, suboptimal drug choice and longer than recommended treatment duration were not associated with the composite poor clinical outcome or CDI. These findings may be related to our definitions of optimal treatment. As our definition of appropriateness of drug choice was based on prior susceptibility results and local antibiograms, it is possible that optimal treatment could of have been overly broad-spectrum coverage to provide sufficient coverage, leading to poor outcomes and CDI.

The only subtype of potentially suboptimal antibiotic treatment that was associated with the composite poor clinical outcome and CDI in our study was potentially suboptimal dose frequency. The risk of CDI was 2.42 times higher among those treated with a suboptimal dose frequency versus optimal treatment. Potentially suboptimal dose frequency was also associated with hospitalization/ED visits and all-cause mortality. In prior work, a dose-dependent increased risk of CDI has been observed, with increasing cumulative defined daily dose of antibiotics resulting in increasing risks of CDI [55]. Our results suggest that dose frequency, in addition to drug choice and antibiotic duration, may also impact risk of CDI and other poor outcomes.

We found no difference in risk for UTI recurrence for residents treated with potentially suboptimal or optimal antibiotic treatment. Previous work has found that among women with UTI, longer courses of antibiotics may be associated with increased risk of recurrent UTI, possibly due to alterations in urogenital flora [56–59]. However, among males with UTI treated in the outpatient setting, longer treatment duration was not associated with UTI recurrence, nor was antibiotic choice [60]. Interestingly, for unclear reasons, among the subgroup of residents treated with initial beta-lactam therapy, suboptimal treatment was associated with a decreased hazard of UTI recurrence which requires further investigation.

It is possible that suboptimal initial treatment could lead to clinical failure, the consequences of which could be serious and may include bacteremia, urosepsis, acute care hospitalization, and potentially death [23, 61]. In our study, suboptimal treatment was not associated with mortality. It is possible that despite being suboptimal, an appropriate agent was ultimately used considering the susceptibility of the uropathogen causing the UTI. Three previous studies have similarly demonstrated that inappropriate empiric therapy for UTI was not associated with an increased risk of mortality [62–64]. Excessive treatment duration versus optimal treatment was associated with a decreased hazard of mortality. The optimal duration of treatment of males with UTI is largely unknown. In females, durations as short as 3 days are recommended for those with uncomplicated infection, but for males longer durations between 7 and 14 days are generally recommended [65–67]. Further investigation is needed to determine optimal treatment duration in males to decrease the risk of treatment failure.

We acknowledge the limitations of this retrospective cohort study. First, our study was not designed to assess causality, and there are important differences (known and unknown) between residents who received suboptimal and optimal antibiotic treatment. While we did adjust for confounders of suboptimal treatment and clinical outcomes, our findings neither confirm nor deny a causal relationship, as our findings may be confounded by unmeasured factors which affect suboptimal treatment and clinical outcomes. As such, while we found an association between suboptimal antibiotic treatment and poor clinical outcomes even after adjusting for several resident and CLC characteristics, this association may be impacted by residual confounding. Second, we defined UTI based on a urine culture and antibiotic treatment, per recommendations of the Infectious Diseases Society of America [65]. While we excluded patients with other positive cultures and only included those exposed to common antibiotics recommended by national guidance or with reliable urinary concentrations, the antibiotic treatment captured may have been targeting another non-urinary tract infection type or asymptomatic bacteriuria. Additionally, we did not capture UTIs in which a urine culture was not obtained. Urine culturing practices are not standardized across VA CLCs, and some facilities may only recommend obtaining UTI cultures in certain clinical situations, such as recurrent infection. However, our population was mostly a complicated, male population, in which urine cultures would be generally recommended. Third, no established definitions for suboptimal antibiotic treatment of residents with UTI have been developed. There is no IDSA guidance for the treatment of complicated UTIs in males, so we based suboptimal treatment definitions on available guidelines for uncomplicated infection in females and expert opinion [65]. Suboptimal antibiotic treatment is multifactorial and as such we considered three elements of suboptimal treatment (drug choice, dose frequency, and longer duration) [25]. However, other elements, such as drug strength and route of administration may also be suboptimal. Insufficient treatment duration is also a clinical problem. We excluded those treated for 2 days or less, but some residents may have still been exposed to insufficient treatment durations. Future research should continue to investigate and operationalize the definition of suboptimal antibiotic treatment among residents with UTI. Additionally, while we conducted analyses by subtype of suboptimal treatment, comparative analyses are warranted to determine which individual aspects of suboptimal antibiotic treatment (the drug choice, dose frequency, or longer than recommended duration) are the most strongly associated with poor outcomes. Forth, we measured all-cause hospitalizations, ED visits, and mortality. Our frail older resident population is already at risk for these events and many of the events observed may have not been related to antibiotic use. Alternatively, we likely underestimated antibiotic-related adverse events, as these events may not have been coded or other codes were used. Adverse events are difficult to identify in large databases as they are often not recorded in health claims and reports [68]. Fifth, while we investigated five potential harms of suboptimal antibiotic use, we did not assess the development of colonization or infection with resistance, which is a well-known harm of antibiotic use. Finally, the generalizability of our findings conducted in a largely older male population may be limited to other non-VA long-term care populations.

## Conclusions

In this large national study of 19,701 CLC residents with an incident UTI, suboptimal antibiotic treatment was associated with increased risk of poor clinical outcomes. Our results were driven by a 94% increased risk of CDI with suboptimal use, meaning not unnecessary use, but not the use of the best drug, dose frequency, and duration. Our work expands on previous work which has found that antibiotic use and unnecessary antibiotic use are associated with a number of harms in vulnerable older long-term care residents. Beyond the decision to use antibiotics, clinicians should consider the potential harms of their antibiotic choices with regards to drug, dose, and duration, to ensure that residents are receiving the most optimal antibiotic treatment. Future research should continue to investigate the impact of suboptimal treatment UTI and other common types of infection among older vulnerable long-term care residents.

## Supplementary Information

**Additional file 1: Table S1.** Sensitivity analyses with varying follow-up times. **Table S2.** Sensitivity analyses using an alternate definition of poor clinical outcome which excludes CDI. **Table S3.** Study definitions used to measure potentially suboptimal antibiotic treatment, and subtypes of potentially suboptimal antibiotic treatment. **Table S4.** Diagnosis and procedure codes used for outcomes definitions.

## Data Availability

The de-identified datasets used and/or analyzed during the current study are available from the corresponding author on reasonable request and approval of the Providence Veterans Affairs Medical Center IRB.

## Abbreviations

CDI: *Clostridioides difficile* infection
CLC: Community Living Center
LTC: Long-term care
LTCF: Long-term care facility
UTI: Urinary Tract Infection
VA: Veterans Affairs
