# Supplementary material for: Poor clinical outcomes associated with suboptimal antibiotic treatment among older long-term care facility residents with urinary tract infection: a retrospective cohort study
Source: BMC Geriatr. 2021 Jul 23;21:436. doi: 10.1186/s12877-021-02378-5 (PMC8299613; doi:10.1186/s12877-021-02378-5)
Supplement: Supplementary file 1 — Additional file 1: Table S1. Sensitivity analyses with varying follow-up times. Table S2. Sensitivity analyses using an alternate definition of poor clinical outcome which excludes CDI. Table S3. Study definitions used to measure potentially suboptimal antibiotic treatment, and subtypes of potentially suboptimal antibiotic treatment. Table S4. Diagnosis and procedure codes used for outcomes definitions. [file 12877_2021_2378_MOESM1_ESM.docx]

**Supplemental Files:**

**TITLE:** Poor clinical outcomes associated with suboptimal antibiotic treatment among older long-term care facility residents with urinary tract infection: A retrospective cohort study

**AUTHORS:** Haley J. Appaneal, PharmD^1,2,3,4^

Theresa I. Shireman, PhD^4^

Vrishali V. Lopes, MS^1^

Vincent Mor, PhD^2,4^

David M. Dosa, MD, MPH^1,2,3,4^

Kerry L. LaPlante, PharmD^1,2,3,5^

Aisling R. Caffrey, PhD, MS^1,2,3,4^

**AFFILIATIONS:**

1. Infectious Diseases Research Program, Providence Veterans Affairs Medical Center, Providence, RI, United States
2. Center of Innovation in Long-Term Support Services, Providence Veterans Affairs Medical Center, Providence, RI, United States
3. College of Pharmacy, University of Rhode Island, Kingston, RI
4. Center for Gerontology & Health Care Research and Department of Health Services Policy & Practice, Brown University School of Public Health, Providence, RI
5. Warren Alpert Medical School of Brown University, Division of Infectious Diseases, Providence, RI

**ADDRESS CORRESPONDENCE:**

Haley J. Appaneal, Pharm.D., Research Health Science Specialist, Providence Veterans Affairs Medical Center, 830 Chalkstone Ave, Providence, RI 02908, 401-273-7100 ext 4150 (office); [haley.appaneal@va.gov](mailto:haley.appaneal@va.gov)

**RUNNING TITLE:** Poor clinical outcomes of UTI treatment

**Supplemental Table 1. Sensitivity analyses with varying follow-up times**

| **Outcome** | **Follow-up** | **Unadjusted HR** | **Lower 95% CI** | **Upper 95% CI** | **Adjusted HR** | **Lower 95% CI** | **Upper 95% CI** |
| --- | --- | --- | --- | --- | --- | --- | --- |
| Poor clinical outcome^a^ | 14 days | 1.10 | 1.03 | 1.17 | **1.10** | **1.04** | **1.17** |
|  | 60 days | 1.02 | 0.98 | 1.06 | 1.03 | 0.99 | 1.07 |
|  | 90 days | 1.01 | 0.97 | 1.04 | 1.02 | 0.98 | 1.05 |
|  | 180 days | 1.00 | 0.96 | 1.03 | 1.01 | 0.97 | 1.04 |
| Hospitalization/ Emergency department visit^b^ | 14 days | 1.07 | 0.98 | 1.17 | 1.09 | 0.99 | 1.19 |
|  | 60 days | 0.97 | 0.92 | 1.03 | 1.00 | 0.95 | 1.06 |
|  | 90 days | 0.95 | 0.91 | 1.00 | 0.99 | 0.94 | 1.04 |
|  | 180 days | 0.96 | 0.92 | 1.01 | 1.00 | 0.96 | 1.05 |
| UTI recurrence^c^ | 14 days | 0.95 | 0.85 | 1.07 | 0.98 | 0.88 | 1.10 |
|  | 60 days | 0.92 | 0.86 | 0.98 | 0.96 | 0.89 | 1.02 |
|  | 90 days | 0.91 | 0.86 | 0.97 | 0.96 | 0.90 | 1.02 |
|  | 180 days | 0.90 | 0.85 | 0.95 | 0.95 | 0.90 | 1.00 |
| All-cause mortality^d^ | 14 days | 0.96 | 0.86 | 1.08 | 0.97 | 0.86 | 1.08 |
|  | 60 days | 1.12 | 1.04 | 1.20 | 1.06 | 0.98 | 1.14 |
|  | 90 days | 1.08 | 1.01 | 1.16 | 1.03 | 0.96 | 1.11 |
|  | 180 days | 1.07 | 1.01 | 1.13 | 1.03 | 0.97 | 1.09 |
| *Clostridioides difficile* infection^e^ | 14 days | 1.20 | 0.91 | 1.57 | 1.33 | 1.00 | 1.77 |
|  | 60 days | 1.80 | 1.49 | 2.17 | **1.74** | **1.44** | **2.11** |
|  | 90 days | 1.61 | 1.36 | 1.91 | **1.56** | **1.32** | **1.85** |
|  | 180 days | 1.46 | 1.26 | 1.69 | **1.43** | **1.23** | **1.66** |
| Antibiotic related adverse drug event^f^ | 14 days | 1.67 | 0.66 | 4.22 | 0.81 | 0.34 | 1.92 |
|  | 60 days | 1.06 | 0.65 | 1.72 | 1.09 | 0.67 | 1.77 |
|  | 90 days | 1.09 | 0.69 | 1.72 | 1.12 | 0.71 | 1.77 |
|  | 180 days | 1.13 | 0.77 | 1.66 | 1.16 | 0.79 | 1.71 |

CI= Confidence interval; CLC= Community Living Center; HR= Hazard ratio; UTI= Urinary tract infection; VAMC= Veterans Affairs Medical Center

Bolded adjusted HRs and 95% CIs indicate statistical significance (p<0.05).

^a^Adjusted for 8 resident-level covariates (genitourinary disease comorbidity, cardiopulmonary comorbidity, chronic renal disease comorbidity, previous fluroquinolone exposure in the past 30 days, age, previous VAMC hospitalization in the past 30 days, previous VAMC urine culture in the past 365 days, high white blood cell count), and 1 CLC-level covariate (total CLC incident UTI rate per 10,000 bed days).

^b^Adjusted for 10 resident-level covariates (cardiopulmonary comorbidity, genitourinary disease comorbidity, chronic renal disease comorbidity, age, previous VAMC hospitalization in the past 30 days, previous outpatient VA urine culture in the past 365 days, previous CLC urine culture in the past 365 days, previous VAMC urine culture in the past 365 days, high white blood cell count, year of episode) and 1 CLC-level covariate (total CLC incident UTI rate per 10,000 bed days).

^c^Adjusted for 5 resident-level covariates (genitourinary disease comorbidity, previous fluroquinolone exposure in the past 30 days, previous fluroquinolone resistant culture in the past 365 days, previous CLC urine culture in the past 365 days, previous VAMC urine culture in the past 365 days), and 1 CLC-level covariate (total CLC incident UTI rate per 10,000 bed days).

^d^Adjusted for 10 resident-level covariates (history of a skin infection diagnosis in the past 365 days, history of a urinary tract infection diagnosis in the past 365 days, chronic renal disease comorbidity, age, previous fluroquinolone exposure in the past 30 days, previous VAMC hospitalization in the past 30 days, previous outpatient VA urine culture in the past 365 days, previous VAMC urine culture in the past 365 days, high white blood cell count, year of episode), and 1 CLC-level covariate (total CLC incident UTI rate per 10,000 bed days).

^e^ Adjusted for 8 resident-level covariates (history of a skin infection diagnosis in the past 365 days, genitourinary disease comorbidity, chronic renal disease comorbidity, previous VAMC hospitalization in the past 30 days, previous CLC urine culture in the past 365 days, previous VAMC urine culture in the past 365 days, high white blood cell count, year of episode), and 1 CLC-level covariate (total CLC incident UTI rate per 10,000 bed days).

^f^Adjusted for 2 resident-level covariates (VAMC hospitalization in the past 30 days, year of episode), and 1 CLC-level covariate (total CLC incident UTI rate per 10,000 bed days).

**Supplemental Table 2. Sensitivity analyses using an alternate definition of poor clinical outcome which excludes CDI**

| **Outcome** | **Follow-up** | **Unadjusted HR** | **Lower 95% CI** | **Upper 95% CI** | **Adjusted HR** | **Lower 95% CI** | **Upper 95% CI** |
| --- | --- | --- | --- | --- | --- | --- | --- |
| Poor clinical outcome, without CDI^a^ | 14 days | 1.08 | 1.01 | 1.15 | **1.08** | **1.01** | **1.15** |
|  | 30 days | 1.03 | 0.98 | 1.08 | 1.03 | 0.98 | 1.09 |
|  | 60 days | 1.00 | 0.96 | 1.04 | 1.01 | 0.97 | 1.05 |
|  | 90 days | 0.99 | 0.95 | 1.03 | 1.00 | 0.96 | 1.04 |
|  | 180 days | 0.99 | 0.95 | 1.02 | 1.00 | 0.96 | 1.03 |

CDI= *Clostridioides difficile* infection; CI= Confidence interval; CLC= Community Living Center; HR= Hazard ratio; UTI= Urinary tract infection; VAMC= Veterans Affairs Medical Center

Bolded adjusted HRs and 95% CIs indicate statistical significance (p<0.05).

Poor clinical outcome, without CDI defined as, UTI recurrence, acute care hospitalization or emergency department (ED) visit, adverse drug event, or death within 30 days of antibiotic discontinuation.

^a^Adjusted for 8 resident-level covariates (genitourinary disease comorbidity, cardiopulmonary comorbidity, chronic renal disease comorbidity, previous fluroquinolone exposure in the past 30 days, age, previous VAMC hospitalization in the past 30 days, previous VAMC urine culture in the past 365 days, high white blood cell count), and 1 CLC-level covariate (total CLC incident UTI rate per 10,000 bed days).

**Supplemental Table 3. Study definitions used to measure potentially suboptimal antibiotic treatment, subtypes of potentially suboptimal antibiotic treatment**

| **Exposure** | **Definition** | **Methods** |
| --- | --- | --- |
| Potentially suboptimal antibiotic treatment | Use of a potentially suboptimal: 1) antibiotic drug choice; 2) antibiotic dose frequency; and/or 3) antibiotic treatment duration. |  |
| Potentially suboptimal antibiotic drug choice | Use of a suboptimal initial antibiotic drug choice on day 1 based on: 1) evidence of resistance to that agent considering the resident’s urine culture and susceptibly (C&S) results from the previous 180 days, or otherwise 2) the local CLC antibiogram (if no previous urine culture and susceptibly results). | If urine C&S results were available in the last 180-days, defined as administration of antibiotic despite previous resistance to that agent.  If no urine C&S results were available in the last 180-days, defined as administration of only antibiotics with insufficient coverage (percent susceptibility <80%) considering the local CLC-urinary antibiogram for the year prior. |
| Potentially suboptimal antibiotic dose frequency | Use of a suboptimal antibiotic dose frequency based on renal function. | The Cockcroft–Gault equation using the most recent serum creatine within the 365 days prior to estimate renal function.  Assessed if the average number of doses per day were concordant with recommended dosage based on renal function.  If the number of doses administered per day were not available, dose frequency was estimated by diving the quantity dispensed by the days’ supply.  If serum creatine was missing dose frequency was defined as suboptimal. |
| Potentially suboptimal treatment duration | Use of a longer than recommended antibiotic treatment duration. | Excessive duration was defined as > 14 days. |

UTI=Urinary tract infection

*Adapted from Appaneal HJ, Caffrey AR, Lopes VV, Dosa DM, Shireman TI, LaPlante KL. Frequency and Predictors of Suboptimal Prescribing Among a Cohort of Older Male Residents with Urinary Tract Infection. Clinical infectious diseases. 2020*

**Supplemental Table 4. Diagnosis and procedure codes used for outcomes definitions**

| **Outcome** | **ICD-9/10 Codes** | **ICD-9/10 Description** |
| --- | --- | --- |
| Hip or knee procedures | 0.7 | REVISION OF HIP REPLACEMENT, BOTH ACETABULAR AND FEMORAL COMPONENTS |
|  | 0.71 | REVISION OF HIP REPLACEMENT, ACETABULAR COMPONENT |
|  | 0.73 | REVISION OF HIP REPLACEMENT, ACETABULAR LINER AND/OR FEMORAL HEAD ONLY |
|  | 0.74 | HIP REPLACEMENT BEARING SURFACE, METAL ON POLYETHYLENE |
|  | 0.75 | HIP REPLACEMENT BEARING SURFACE, METAL-ON-METAL |
|  | 0.76 | HIP REPLACEMENT BEARING SURFACE, CERAMIC-ON-CERAMIC |
|  | 0.77 | HIP REPLACEMENT BEARING SURFACE, CERAMIC-ON-POLYETHYLENE |
|  | 0.8 | REVISION OF KNEE REPLACEMENT, TOTAL (ALL COMPONENTS) |
|  | 0.81 | REVISION OF KNEE REPLACEMENT, TIBIAL COMPONENT |
|  | 0.82 | REVISION OF KNEE REPLACEMENT, FEMORAL COMPONENT |
|  | 0.83 | REVISION OF KNEE REPLACEMENT, PATELLAR COMPONENT |
|  | 0.84 | REVISION OF TOTAL KNEE REPLACEMENT, TIBIAL INSERT (LINER) |
|  | 80.05 | ARTHROTOMY FOR REMOVAL OF PROSTHESIS WITHOUT REPLACEMENT, HIP |
|  | 80.06 | ARTHROTOMY FOR REMOVAL OF PROSTHESIS WITHOUT REPLACEMENT, KNEE |
|  | 81.51 | TOTAL HIP REPLACEMENT |
|  | 81.52 | PARTIAL HIP REPLACEMENT |
|  | 81.53 | REVISION OF HIP REPLACEMENT |
|  | 81.54 | TOTAL KNEE REPLACEMENT |
|  | 81.55 | REVISION OF KNEE REPLACEMENT |
|  | 0MRM07Z | REPLACEMENT OF LEFT HIP BURSA AND LIGAMENT WITH AUTOLOGOUS TISSUE SUBSTITUTE, OPEN APPROACH |
|  | 0SR901A | REPLACEMENT OF RIGHT HIP JOINT WITH METAL SYNTHETIC SUBSTITUTE, UNCEMENTED, OPEN APPROACH |
|  | 0SR901Z | REPLACEMENT OF RIGHT HIP JOINT WITH METAL SYNTHETIC SUBSTITUTE, OPEN APPROACH |
|  | 0SR9029 | REPLACEMENT OF RIGHT HIP JOINT WITH METAL ON POLYETHYLENE SYNTHETIC SUBSTITUTE, CEMENTED, OPEN APPROACH |
|  | 0SR902A | REPLACEMENT OF RIGHT HIP JOINT WITH METAL ON POLYETHYLENE SYNTHETIC SUBSTITUTE, UNCEMENTED, OPEN APPROACH |
|  | 0SR902Z | REPLACEMENT OF RIGHT HIP JOINT WITH METAL ON POLYETHYLENE SYNTHETIC SUBSTITUTE, OPEN APPROACH |
|  | 0SR903A | REPLACEMENT OF RIGHT HIP JOINT WITH CERAMIC SYNTHETIC SUBSTITUTE, UNCEMENTED, OPEN APPROACH |
|  | 0SR903Z | REPLACEMENT OF RIGHT HIP JOINT WITH CERAMIC SYNTHETIC SUBSTITUTE, OPEN APPROACH |
|  | 0SR904A | REPLACEMENT OF RIGHT HIP JOINT WITH CERAMIC ON POLYETHYLENE SYNTHETIC SUBSTITUTE, UNCEMENTED, OPEN APPROACH |
|  | 0SR904Z | REPLACEMENT OF RIGHT HIP JOINT WITH CERAMIC ON POLYETHYLENE SYNTHETIC SUBSTITUTE, OPEN APPROACH |
|  | 0SR906A | REPLACEMENT OF RIGHT HIP JOINT WITH OXIDIZED ZIRCONIUM ON POLYETHYLENE SYNTHETIC SUBSTITUTE, UNCEMENTED, OPEN APPROACH |
|  | 0SR906Z | REPLACEMENT OF RIGHT HIP JOINT WITH OXIDIZED ZIRCONIUM ON POLYETHYLENE SYNTHETIC SUBSTITUTE, OPEN APPROACH |
|  | 0SR90J9 | REPLACEMENT OF RIGHT HIP JOINT WITH SYNTHETIC SUBSTITUTE, CEMENTED, OPEN APPROACH |
|  | 0SR90JA | REPLACEMENT OF RIGHT HIP JOINT WITH SYNTHETIC SUBSTITUTE, UNCEMENTED, OPEN APPROACH |
|  | 0SR90JZ | REPLACEMENT OF RIGHT HIP JOINT WITH SYNTHETIC SUBSTITUTE, OPEN APPROACH |
|  | 0SR90KZ | REPLACEMENT OF RIGHT HIP JOINT WITH NONAUTOLOGOUS TISSUE SUBSTITUTE, OPEN APPROACH |
|  | 0SRA00A | REPLACEMENT OF RIGHT HIP JOINT, ACETABULAR SURFACE WITH POLYETHYLENE SYNTHETIC SUBSTITUTE, UNCEMENTED, OPEN APPROACH |
|  | 0SRA00Z | REPLACEMENT OF RIGHT HIP JOINT, ACETABULAR SURFACE WITH POLYETHYLENE SYNTHETIC SUBSTITUTE, OPEN APPROACH |
|  | 0SRA01A | REPLACEMENT OF RIGHT HIP JOINT, ACETABULAR SURFACE WITH METAL SYNTHETIC SUBSTITUTE, UNCEMENTED, OPEN APPROACH |
|  | 0SRA03A | REPLACEMENT OF RIGHT HIP JOINT, ACETABULAR SURFACE WITH CERAMIC SYNTHETIC SUBSTITUTE, UNCEMENTED, OPEN APPROACH |
|  | 0SRA0J9 | REPLACEMENT OF RIGHT HIP JOINT, ACETABULAR SURFACE WITH SYNTHETIC SUBSTITUTE, CEMENTED, OPEN APPROACH |
|  | 0SRB01Z | REPLACEMENT OF LEFT HIP JOINT WITH METAL SYNTHETIC SUBSTITUTE, OPEN APPROACH |
|  | 0SRB029 | REPLACEMENT OF LEFT HIP JOINT WITH METAL ON POLYETHYLENE SYNTHETIC SUBSTITUTE, CEMENTED, OPEN APPROACH |
|  | 0SRB02A | REPLACEMENT OF LEFT HIP JOINT WITH METAL ON POLYETHYLENE SYNTHETIC SUBSTITUTE, UNCEMENTED, OPEN APPROACH |
|  | 0SRB02Z | REPLACEMENT OF LEFT HIP JOINT WITH METAL ON POLYETHYLENE SYNTHETIC SUBSTITUTE, OPEN APPROACH |
|  | 0SRB03A | REPLACEMENT OF LEFT HIP JOINT WITH CERAMIC SYNTHETIC SUBSTITUTE, UNCEMENTED, OPEN APPROACH |
|  | 0SRB03Z | REPLACEMENT OF LEFT HIP JOINT WITH CERAMIC SYNTHETIC SUBSTITUTE, OPEN APPROACH |
|  | 0SRB049 | REPLACEMENT OF LEFT HIP JOINT WITH CERAMIC ON POLYETHYLENE SYNTHETIC SUBSTITUTE, CEMENTED, OPEN APPROACH |
|  | 0SRB04A | REPLACEMENT OF LEFT HIP JOINT WITH CERAMIC ON POLYETHYLENE SYNTHETIC SUBSTITUTE, UNCEMENTED, OPEN APPROACH |
|  | 0SRB04Z | REPLACEMENT OF LEFT HIP JOINT WITH CERAMIC ON POLYETHYLENE SYNTHETIC SUBSTITUTE, OPEN APPROACH |
|  | 0SRB069 | REPLACEMENT OF LEFT HIP JOINT WITH OXIDIZED ZIRCONIUM ON POLYETHYLENE SYNTHETIC SUBSTITUTE, CEMENTED, OPEN APPROACH |
|  | 0SRB0J9 | REPLACEMENT OF LEFT HIP JOINT WITH SYNTHETIC SUBSTITUTE, CEMENTED, OPEN APPROACH |
|  | 0SRB0JA | REPLACEMENT OF LEFT HIP JOINT WITH SYNTHETIC SUBSTITUTE, UNCEMENTED, OPEN APPROACH |
|  | 0SRB0JZ | REPLACEMENT OF LEFT HIP JOINT WITH SYNTHETIC SUBSTITUTE, OPEN APPROACH |
|  | 0SRB0KZ | REPLACEMENT OF LEFT HIP JOINT WITH NONAUTOLOGOUS TISSUE SUBSTITUTE, OPEN APPROACH |
|  | 0SRC069 | REPLACEMENT OF RIGHT KNEE JOINT WITH OXIDIZED ZIRCONIUM ON POLYETHYLENE SYNTHETIC SUBSTITUTE, CEMENTED, OPEN APPROACH |
|  | 0SRC07Z | REPLACEMENT OF RIGHT KNEE JOINT WITH AUTOLOGOUS TISSUE SUBSTITUTE, OPEN APPROACH |
|  | 0SRC0EZ | REPLACEMENT OF RIGHT KNEE JOINT WITH ARTICULATING SPACER, OPEN APPROACH |
|  | 0SRC0J9 | REPLACEMENT OF RIGHT KNEE JOINT WITH SYNTHETIC SUBSTITUTE, CEMENTED, OPEN APPROACH |
|  | 0SRC0JA | REPLACEMENT OF RIGHT KNEE JOINT WITH SYNTHETIC SUBSTITUTE, UNCEMENTED, OPEN APPROACH |
|  | 0SRC0JZ | REPLACEMENT OF RIGHT KNEE JOINT WITH SYNTHETIC SUBSTITUTE, OPEN APPROACH |
|  | 0SRC0KZ | REPLACEMENT OF RIGHT KNEE JOINT WITH NONAUTOLOGOUS TISSUE SUBSTITUTE, OPEN APPROACH |
|  | 0SRC0L9 | REPLACEMENT OF RIGHT KNEE JOINT WITH MEDIAL UNICONDYLAR SYNTHETIC SUBSTITUTE, CEMENTED, OPEN APPROACH |
|  | 0SRC0N9 | REPLACEMENT OF RIGHT KNEE JOINT WITH PATELLOFEMORAL SYNTHETIC SUBSTITUTE, CEMENTED, OPEN APPROACH |
|  | 0SRD069 | REPLACEMENT OF LEFT KNEE JOINT WITH OXIDIZED ZIRCONIUM ON POLYETHYLENE SYNTHETIC SUBSTITUTE, CEMENTED, OPEN APPROACH |
|  | 0SRD07Z | REPLACEMENT OF LEFT KNEE JOINT WITH AUTOLOGOUS TISSUE SUBSTITUTE, OPEN APPROACH |
|  | 0SRD0J9 | REPLACEMENT OF LEFT KNEE JOINT WITH SYNTHETIC SUBSTITUTE, CEMENTED, OPEN APPROACH |
|  | 0SRD0JZ | REPLACEMENT OF LEFT KNEE JOINT WITH SYNTHETIC SUBSTITUTE, OPEN APPROACH |
|  | 0SRD0KZ | REPLACEMENT OF LEFT KNEE JOINT WITH NONAUTOLOGOUS TISSUE SUBSTITUTE, OPEN APPROACH |
|  | 0SRD0L9 | REPLACEMENT OF LEFT KNEE JOINT WITH MEDIAL UNICONDYLAR SYNTHETIC SUBSTITUTE, CEMENTED, OPEN APPROACH |
|  | 0SRD0LZ | REPLACEMENT OF LEFT KNEE JOINT WITH MEDIAL UNICONDYLAR SYNTHETIC SUBSTITUTE, OPEN APPROACH |
|  | 0SRD0N9 | REPLACEMENT OF LEFT KNEE JOINT WITH PATELLOFEMORAL SYNTHETIC SUBSTITUTE, CEMENTED, OPEN APPROACH |
|  | 0SRD0NA | REPLACEMENT OF LEFT KNEE JOINT WITH PATELLOFEMORAL SYNTHETIC SUBSTITUTE, UNCEMENTED, OPEN APPROACH |
|  | 0SRE009 | REPLACEMENT OF LEFT HIP JOINT, ACETABULAR SURFACE WITH POLYETHYLENE SYNTHETIC SUBSTITUTE, CEMENTED, OPEN APPROACH |
|  | 0SRE01A | REPLACEMENT OF LEFT HIP JOINT, ACETABULAR SURFACE WITH METAL SYNTHETIC SUBSTITUTE, UNCEMENTED, OPEN APPROACH |
|  | 0SRE0KZ | REPLACEMENT OF LEFT HIP JOINT, ACETABULAR SURFACE WITH NONAUTOLOGOUS TISSUE SUBSTITUTE, OPEN APPROACH |
|  | 0SRR01A | REPLACEMENT OF RIGHT HIP JOINT, FEMORAL SURFACE WITH METAL SYNTHETIC SUBSTITUTE, UNCEMENTED, OPEN APPROACH |
|  | 0SRR01Z | REPLACEMENT OF RIGHT HIP JOINT, FEMORAL SURFACE WITH METAL SYNTHETIC SUBSTITUTE, OPEN APPROACH |
|  | 0SRR03A | REPLACEMENT OF RIGHT HIP JOINT, FEMORAL SURFACE WITH CERAMIC SYNTHETIC SUBSTITUTE, UNCEMENTED, OPEN APPROACH |
|  | 0SRR03Z | REPLACEMENT OF RIGHT HIP JOINT, FEMORAL SURFACE WITH CERAMIC SYNTHETIC SUBSTITUTE, OPEN APPROACH |
|  | 0SRR0JA | REPLACEMENT OF RIGHT HIP JOINT, FEMORAL SURFACE WITH SYNTHETIC SUBSTITUTE, UNCEMENTED, OPEN APPROACH |
|  | 0SRR0JZ | REPLACEMENT OF RIGHT HIP JOINT, FEMORAL SURFACE WITH SYNTHETIC SUBSTITUTE, OPEN APPROACH |
|  | 0SRS01A | REPLACEMENT OF LEFT HIP JOINT, FEMORAL SURFACE WITH METAL SYNTHETIC SUBSTITUTE, UNCEMENTED, OPEN APPROACH |
|  | 0SRS03Z | REPLACEMENT OF LEFT HIP JOINT, FEMORAL SURFACE WITH CERAMIC SYNTHETIC SUBSTITUTE, OPEN APPROACH |
|  | 0SRS0J9 | REPLACEMENT OF LEFT HIP JOINT, FEMORAL SURFACE WITH SYNTHETIC SUBSTITUTE, CEMENTED, OPEN APPROACH |
|  | 0SRS0JZ | REPLACEMENT OF LEFT HIP JOINT, FEMORAL SURFACE WITH SYNTHETIC SUBSTITUTE, OPEN APPROACH |
|  | 0SRT0JZ | REPLACEMENT OF RIGHT KNEE JOINT, FEMORAL SURFACE WITH SYNTHETIC SUBSTITUTE, OPEN APPROACH |
|  | 0SRT0KZ | REPLACEMENT OF RIGHT KNEE JOINT, FEMORAL SURFACE WITH NONAUTOLOGOUS TISSUE SUBSTITUTE, OPEN APPROACH |
|  | 0SRU0J9 | REPLACEMENT OF LEFT KNEE JOINT, FEMORAL SURFACE WITH SYNTHETIC SUBSTITUTE, CEMENTED, OPEN APPROACH |
|  | 0SRU0JA | REPLACEMENT OF LEFT KNEE JOINT, FEMORAL SURFACE WITH SYNTHETIC SUBSTITUTE, UNCEMENTED, OPEN APPROACH |
|  | 0SRW0J9 | REPLACEMENT OF LEFT KNEE JOINT, TIBIAL SURFACE WITH SYNTHETIC SUBSTITUTE, CEMENTED, OPEN APPROACH |
| Antibiotic related adverse drug event^f^ | 960 | POISONING BY PENICILLINS |
|  | 960.1 | POISONING BY ANTIFUNGAL ANTIBIOTICS |
|  | 960.2 | POISONING BY CHLORAMPHENICOL GROUP |
|  | 960.3 | POISONING BY ERYTHROMYCIN AND OTHER MACROLIDES |
|  | 960.4 | POISONING BY TETRACYCLINE GROUP |
|  | 960.5 | POISONING OF CEPHALOSPORIN GROUP |
|  | 960.8 | POISONING BY OTHER SPECIFIED ANTIBIOTICS |
|  | 960.9 | POISONING BY UNSPECIFIED ANTIBIOTIC |
|  | E930.0 | PENICILLINS CAUSING ADVERSE EFFECTS IN THERAPEUTIC USE |
|  | E930.1 | ANTIFUNGAL ANTIBIOTICS CAUSING ADVERSE EFFECTS IN THERAPEUTIC USE |
|  | E930.2 | CHLORAMPHENICOL GROUP CAUSING ADVERSE EFFECTS IN THERAPEUTIC USE |
|  | E930.3 | ERYTHROMYCIN AND OTHER MACROLIDES CAUSING ADVERSE EFFECTS IN THERAPEUTIC USE |
|  | E930.4 | TETRACYCLINE GROUP CAUSING ADVERSE EFFECTS IN THERAPEUTIC USE |
|  | E930.5 | CEPHALOSPORIN GROUP CAUSING ADVERSE EFFECTS IN THERAPEUTIC USE |
|  | E930.6 | ANTIMYCOBACTERIAL ANTIBIOTICS CAUSING ADVERSE EFFECTS IN THERAPEUTIC USE |
|  | E930.8 | OTHER SPECIFIED ANTIBIOTICS CAUSING ADVERSE EFFECTS IN THERAPEUTIC USE |
|  | T36.0X1A | POISONING BY PENICILLINS, ACCIDENTAL (UNINTENTIONAL), INITIAL ENCOUNTER |
|  | T36.0X1D | POISONING BY PENICILLINS, ACCIDENTAL (UNINTENTIONAL), SUBSEQUENT ENCOUNTER |
|  | T36.0X1S | POISONING BY PENICILLINS, ACCIDENTAL (UNINTENTIONAL), SEQUELA |
|  | T36.0X2A | POISONING BY PENICILLINS, INTENTIONAL SELF-HARM, INITIAL ENCOUNTER |
|  | T36.0X2D | POISONING BY PENICILLINS, INTENTIONAL SELF-HARM, SUBSEQUENT ENCOUNTER |
|  | T36.0X2S | POISONING BY PENICILLINS, INTENTIONAL SELF-HARM, SEQUELA |
|  | T36.0X3A | POISONING BY PENICILLINS, ASSAULT, INITIAL ENCOUNTER |
|  | T36.0X3D | POISONING BY PENICILLINS, ASSAULT, SUBSEQUENT ENCOUNTER |
|  | T36.0X3S | POISONING BY PENICILLINS, ASSAULT, SEQUELA |
|  | T36.0X4A | POISONING BY PENICILLINS, UNDETERMINED, INITIAL ENCOUNTER |
|  | T36.0X4D | POISONING BY PENICILLINS, UNDETERMINED, SUBSEQUENT ENCOUNTER |
|  | T36.0X4S | POISONING BY PENICILLINS, UNDETERMINED, SEQUELA |
|  | T36.0X5A | ADVERSE EFFECT OF PENICILLINS, INITIAL ENCOUNTER |
|  | T36.0X5D | ADVERSE EFFECT OF PENICILLINS, SUBSEQUENT ENCOUNTER |
|  | T36.0X5S | ADVERSE EFFECT OF PENICILLINS, SEQUELA |
|  | T36.0X6A | UNDERDOSING OF PENICILLINS, INITIAL ENCOUNTER |
|  | T36.0X6D | UNDERDOSING OF PENICILLINS, SUBSEQUENT ENCOUNTER |
|  | T36.0X6S | UNDERDOSING OF PENICILLINS, SEQUELA |
|  | T36.1X1A | POISONING BY CEPHALOSPORINS AND OTHER BETA-LACTAM ANTIBIOTICS, ACCIDENTAL (UNINTENTIONAL), INITIAL ENCOUNTER |
|  | T36.1X1D | POISONING BY CEPHALOSPORINS AND OTHER BETA-LACTAM ANTIBIOTICS, ACCIDENTAL (UNINTENTIONAL), SUBSEQUENT ENCOUNTER |
|  | T36.1X1S | POISONING BY CEPHALOSPORINS AND OTHER BETA-LACTAM ANTIBIOTICS, ACCIDENTAL (UNINTENTIONAL), SEQUELA |
|  | T36.1X2A | POISONING BY CEPHALOSPORINS AND OTHER BETA-LACTAM ANTIBIOTICS, INTENTIONAL SELF-HARM, INITIAL ENCOUNTER |
|  | T36.1X2D | POISONING BY CEPHALOSPORINS AND OTHER BETA-LACTAM ANTIBIOTICS, INTENTIONAL SELF-HARM, SUBSEQUENT ENCOUNTER |
|  | T36.1X2S | POISONING BY CEPHALOSPORINS AND OTHER BETA-LACTAM ANTIBIOTICS, INTENTIONAL SELF-HARM, SEQUELA |
|  | T36.1X3A | POISONING BY CEPHALOSPORINS AND OTHER BETA-LACTAM ANTIBIOTICS, ASSAULT, INITIAL ENCOUNTER |
|  | T36.1X3D | POISONING BY CEPHALOSPORINS AND OTHER BETA-LACTAM ANTIBIOTICS, ASSAULT, SUBSEQUENT ENCOUNTER |
|  | T36.1X3S | POISONING BY CEPHALOSPORINS AND OTHER BETA-LACTAM ANTIBIOTICS, ASSAULT, SEQUELA |
|  | T36.1X4A | POISONING BY CEPHALOSPORINS AND OTHER BETA-LACTAM ANTIBIOTICS, UNDETERMINED, INITIAL ENCOUNTER |
|  | T36.1X4D | POISONING BY CEPHALOSPORINS AND OTHER BETA-LACTAM ANTIBIOTICS, UNDETERMINED, SUBSEQUENT ENCOUNTER |
|  | T36.1X4S | POISONING BY CEPHALOSPORINS AND OTHER BETA-LACTAM ANTIBIOTICS, UNDETERMINED, SEQUELA |
|  | T36.1X5A | ADVERSE EFFECT OF CEPHALOSPORINS AND OTHER BETA-LACTAM ANTIBIOTICS, INITIAL ENCOUNTER |
|  | T36.1X5D | ADVERSE EFFECT OF CEPHALOSPORINS AND OTHER BETA-LACTAM ANTIBIOTICS, SUBSEQUENT ENCOUNTER |
|  | T36.1X5S | ADVERSE EFFECT OF CEPHALOSPORINS AND OTHER BETA-LACTAM ANTIBIOTICS, SEQUELA |
|  | T36.1X6A | UNDERDOSING OF CEPHALOSPORINS AND OTHER BETA-LACTAM ANTIBIOTICS, INITIAL ENCOUNTER |
|  | T36.1X6D | UNDERDOSING OF CEPHALOSPORINS AND OTHER BETA-LACTAM ANTIBIOTICS, SUBSEQUENT ENCOUNTER |
|  | T36.2X1A | POISONING BY CHLORAMPHENICOL GROUP, ACCIDENTAL (UNINTENTIONAL), INITIAL ENCOUNTER |
|  | T36.2X1D | POISONING BY CHLORAMPHENICOL GROUP, ACCIDENTAL (UNINTENTIONAL), SUBSEQUENT ENCOUNTER |
|  | T36.2X1S | POISONING BY CHLORAMPHENICOL GROUP, ACCIDENTAL (UNINTENTIONAL), SEQUELA |
|  | T36.2X2A | POISONING BY CHLORAMPHENICOL GROUP, INTENTIONAL SELF-HARM, INITIAL ENCOUNTER |
|  | T36.2X2D | POISONING BY CHLORAMPHENICOL GROUP, INTENTIONAL SELF-HARM, SUBSEQUENT ENCOUNTER |
|  | T36.2X2S | POISONING BY CHLORAMPHENICOL GROUP, INTENTIONAL SELF-HARM, SEQUELA |
|  | T36.2X3A | POISONING BY CHLORAMPHENICOL GROUP, ASSAULT, INITIAL ENCOUNTER |
|  | T36.2X3D | POISONING BY CHLORAMPHENICOL GROUP, ASSAULT, SUBSEQUENT ENCOUNTER |
|  | T36.2X3S | POISONING BY CHLORAMPHENICOL GROUP, ASSAULT, SEQUELA |
|  | T36.2X4A | POISONING BY CHLORAMPHENICOL GROUP, UNDETERMINED, INITIAL ENCOUNTER |
|  | T36.2X4D | POISONING BY CHLORAMPHENICOL GROUP, UNDETERMINED, SUBSEQUENT ENCOUNTER |
|  | T36.2X4S | POISONING BY CHLORAMPHENICOL GROUP, UNDETERMINED, SEQUELA |
|  | T36.2X5A | ADVERSE EFFECT OF CHLORAMPHENICOL GROUP, INITIAL ENCOUNTER |
|  | T36.2X5D | ADVERSE EFFECT OF CHLORAMPHENICOL GROUP, SUBSEQUENT ENCOUNTER |
|  | T36.2X5S | ADVERSE EFFECT OF CHLORAMPHENICOL GROUP, SEQUELA |
|  | T36.2X6A | UNDERDOSING OF CHLORAMPHENICOL GROUP, INITIAL ENCOUNTER |
|  | T36.2X6D | UNDERDOSING OF CHLORAMPHENICOL GROUP, SUBSEQUENT ENCOUNTER |
|  | T36.2X6S | UNDERDOSING OF CHLORAMPHENICOL GROUP, SEQUELA |
|  | T36.3X1A | POISONING BY MACROLIDES, ACCIDENTAL (UNINTENTIONAL), INITIAL ENCOUNTER |
|  | T36.3X1D | POISONING BY MACROLIDES, ACCIDENTAL (UNINTENTIONAL), SUBSEQUENT ENCOUNTER |
|  | T36.3X1S | POISONING BY MACROLIDES, ACCIDENTAL (UNINTENTIONAL), SEQUELA |
|  | T36.3X2A | POISONING BY MACROLIDES, INTENTIONAL SELF-HARM, INITIAL ENCOUNTER |
|  | T36.3X2D | POISONING BY MACROLIDES, INTENTIONAL SELF-HARM, SUBSEQUENT ENCOUNTER |
|  | T36.3X2S | POISONING BY MACROLIDES, INTENTIONAL SELF-HARM, SEQUELA |
|  | T36.3X3A | POISONING BY MACROLIDES, ASSAULT, INITIAL ENCOUNTER |
|  | T36.3X3D | POISONING BY MACROLIDES, ASSAULT, SUBSEQUENT ENCOUNTER |
|  | T36.3X3S | POISONING BY MACROLIDES, ASSAULT, SEQUELA |
|  | T36.3X4A | POISONING BY MACROLIDES, UNDETERMINED, INITIAL ENCOUNTER |
|  | T36.3X4D | POISONING BY MACROLIDES, UNDETERMINED, SUBSEQUENT ENCOUNTER |
|  | T36.3X4S | POISONING BY MACROLIDES, UNDETERMINED, SEQUELA |
|  | T36.3X5A | ADVERSE EFFECT OF MACROLIDES, INITIAL ENCOUNTER |
|  | T36.3X5D | ADVERSE EFFECT OF MACROLIDES, SUBSEQUENT ENCOUNTER |
|  | T36.3X5S | ADVERSE EFFECT OF MACROLIDES, SEQUELA |
|  | T36.3X6A | UNDERDOSING OF MACROLIDES, INITIAL ENCOUNTER |
|  | T36.3X6D | UNDERDOSING OF MACROLIDES, SUBSEQUENT ENCOUNTER |
|  | T36.3X6S | UNDERDOSING OF MACROLIDES, SEQUELA |
|  | T36.4X1A | POISONING BY TETRACYCLINES, ACCIDENTAL (UNINTENTIONAL), INITIAL ENCOUNTER |
|  | T36.4X1D | POISONING BY TETRACYCLINES, ACCIDENTAL (UNINTENTIONAL), SUBSEQUENT ENCOUNTER |
|  | T36.4X1S | POISONING BY TETRACYCLINES, ACCIDENTAL (UNINTENTIONAL), SEQUELA |
|  | T36.4X2A | POISONING BY TETRACYCLINES, INTENTIONAL SELF-HARM, INITIAL ENCOUNTER |
|  | T36.4X2D | POISONING BY TETRACYCLINES, INTENTIONAL SELF-HARM, SUBSEQUENT ENCOUNTER |
|  | T36.4X3A | POISONING BY TETRACYCLINES, ASSAULT, INITIAL ENCOUNTER |
|  | T36.4X3D | POISONING BY TETRACYCLINES, ASSAULT, SUBSEQUENT ENCOUNTER |
|  | T36.4X3S | POISONING BY TETRACYCLINES, ASSAULT, SEQUELA |
|  | T36.4X4A | POISONING BY TETRACYCLINES, UNDETERMINED, INITIAL ENCOUNTER |
|  | T36.4X4D | POISONING BY TETRACYCLINES, UNDETERMINED, SUBSEQUENT ENCOUNTER |
|  | T36.4X4S | POISONING BY TETRACYCLINES, UNDETERMINED, SEQUELA |
|  | T36.4X5A | ADVERSE EFFECT OF TETRACYCLINES, INITIAL ENCOUNTER |
|  | T36.4X5D | ADVERSE EFFECT OF TETRACYCLINES, SUBSEQUENT ENCOUNTER |
|  | T36.4X5S | ADVERSE EFFECT OF TETRACYCLINES, SEQUELA |
|  | T36.4X6A | UNDERDOSING OF TETRACYCLINES, INITIAL ENCOUNTER |
|  | T36.4X6D | UNDERDOSING OF TETRACYCLINES, SUBSEQUENT ENCOUNTER |
|  | T36.4X6S | UNDERDOSING OF TETRACYCLINES, SEQUELA |
|  | T36.5X1A | POISONING BY AMINOGLYCOSIDES, ACCIDENTAL (UNINTENTIONAL), INITIAL ENCOUNTER |
|  | T36.5X1D | POISONING BY AMINOGLYCOSIDES, ACCIDENTAL (UNINTENTIONAL), SUBSEQUENT ENCOUNTER |
|  | T36.5X1S | POISONING BY AMINOGLYCOSIDES, ACCIDENTAL (UNINTENTIONAL), SEQUELA |
|  | T36.5X2A | POISONING BY AMINOGLYCOSIDES, INTENTIONAL SELF-HARM, INITIAL ENCOUNTER |
|  | T36.5X2D | POISONING BY AMINOGLYCOSIDES, INTENTIONAL SELF-HARM, SUBSEQUENT ENCOUNTER |
|  | T36.5X2S | POISONING BY AMINOGLYCOSIDES, INTENTIONAL SELF-HARM, SEQUELA |
|  | T36.5X3A | POISONING BY AMINOGLYCOSIDES, ASSAULT, INITIAL ENCOUNTER |
|  | T36.5X3D | POISONING BY AMINOGLYCOSIDES, ASSAULT, SUBSEQUENT ENCOUNTER |
|  | T36.5X3S | POISONING BY AMINOGLYCOSIDES, ASSAULT, SEQUELA |
|  | T36.5X4A | POISONING BY AMINOGLYCOSIDES, UNDETERMINED, INITIAL ENCOUNTER |
|  | T36.5X4D | POISONING BY AMINOGLYCOSIDES, UNDETERMINED, SUBSEQUENT ENCOUNTER |
|  | T36.5X4S | POISONING BY AMINOGLYCOSIDES, UNDETERMINED, SEQUELA |
|  | T36.5X5A | ADVERSE EFFECT OF AMINOGLYCOSIDES, INITIAL ENCOUNTER |
|  | T36.5X5D | ADVERSE EFFECT OF AMINOGLYCOSIDES, SUBSEQUENT ENCOUNTER |
|  | T36.5X6A | UNDERDOSING OF AMINOGLYCOSIDES, INITIAL ENCOUNTER |
|  | T36.5X6D | UNDERDOSING OF AMINOGLYCOSIDES, SUBSEQUENT ENCOUNTER |
|  | T36.5X6S | UNDERDOSING OF AMINOGLYCOSIDES, SEQUELA |
|  | T36.6X1A | POISONING BY RIFAMPICINS, ACCIDENTAL (UNINTENTIONAL), INITIAL ENCOUNTER |
|  | T36.6X1D | POISONING BY RIFAMPICINS, ACCIDENTAL (UNINTENTIONAL), SUBSEQUENT ENCOUNTER |
|  | T36.6X1S | POISONING BY RIFAMPICINS, ACCIDENTAL (UNINTENTIONAL), SEQUELA |
|  | T36.6X2A | POISONING BY RIFAMPICINS, INTENTIONAL SELF-HARM, INITIAL ENCOUNTER |
|  | T36.6X2D | POISONING BY RIFAMPICINS, INTENTIONAL SELF-HARM, SUBSEQUENT ENCOUNTER |
|  | T36.6X2S | POISONING BY RIFAMPICINS, INTENTIONAL SELF-HARM, SEQUELA |
|  | T36.6X3A | POISONING BY RIFAMPICINS, ASSAULT, INITIAL ENCOUNTER |
|  | T36.6X3D | POISONING BY RIFAMPICINS, ASSAULT, SUBSEQUENT ENCOUNTER |
|  | T36.6X3S | POISONING BY RIFAMPICINS, ASSAULT, SEQUELA |
|  | T36.6X4A | POISONING BY RIFAMPICINS, UNDETERMINED, INITIAL ENCOUNTER |
|  | T36.6X4D | POISONING BY RIFAMPICINS, UNDETERMINED, SUBSEQUENT ENCOUNTER |
|  | T36.6X4S | POISONING BY RIFAMPICINS, UNDETERMINED, SEQUELA |
|  | T36.6X5A | ADVERSE EFFECT OF RIFAMPICINS, INITIAL ENCOUNTER |
|  | T36.6X5D | ADVERSE EFFECT OF RIFAMPICINS, SUBSEQUENT ENCOUNTER |
|  | T36.6X5S | ADVERSE EFFECT OF RIFAMPICINS, SEQUELA |
|  | T36.6X6A | UNDERDOSING OF RIFAMPICINS, INITIAL ENCOUNTER |
|  | T36.6X6D | UNDERDOSING OF RIFAMPICINS, SUBSEQUENT ENCOUNTER |
|  | T36.6X6S | UNDERDOSING OF RIFAMPICINS, SEQUELA |
|  | T36.7X1A | POISONING BY ANTIFUNGAL ANTIBIOTICS, SYSTEMICALLY USED, ACCIDENTAL (UNINTENTIONAL), INITIAL ENCOUNTER |
|  | T36.7X1D | POISONING BY ANTIFUNGAL ANTIBIOTICS, SYSTEMICALLY USED, ACCIDENTAL (UNINTENTIONAL), SUBSEQUENT ENCOUNTER |
|  | T36.7X1S | POISONING BY ANTIFUNGAL ANTIBIOTICS, SYSTEMICALLY USED, ACCIDENTAL (UNINTENTIONAL), SEQUELA |
|  | T36.7X2A | POISONING BY ANTIFUNGAL ANTIBIOTICS, SYSTEMICALLY USED, INTENTIONAL SELF-HARM, INITIAL ENCOUNTER |
|  | T36.7X2D | POISONING BY ANTIFUNGAL ANTIBIOTICS, SYSTEMICALLY USED, INTENTIONAL SELF-HARM, SUBSEQUENT ENCOUNTER |
|  | T36.7X2S | POISONING BY ANTIFUNGAL ANTIBIOTICS, SYSTEMICALLY USED, INTENTIONAL SELF-HARM, SEQUELA |
|  | T36.7X3A | POISONING BY ANTIFUNGAL ANTIBIOTICS, SYSTEMICALLY USED, ASSAULT, INITIAL ENCOUNTER |
|  | T36.7X3D | POISONING BY ANTIFUNGAL ANTIBIOTICS, SYSTEMICALLY USED, ASSAULT, SUBSEQUENT ENCOUNTER |
|  | T36.7X3S | POISONING BY ANTIFUNGAL ANTIBIOTICS, SYSTEMICALLY USED, ASSAULT, SEQUELA |
|  | T36.7X4A | POISONING BY ANTIFUNGAL ANTIBIOTICS, SYSTEMICALLY USED, UNDETERMINED, INITIAL ENCOUNTER |
|  | T36.7X4D | POISONING BY ANTIFUNGAL ANTIBIOTICS, SYSTEMICALLY USED, UNDETERMINED, SUBSEQUENT ENCOUNTER |
|  | T36.7X4S | POISONING BY ANTIFUNGAL ANTIBIOTICS, SYSTEMICALLY USED, UNDETERMINED, SEQUELA |
|  | T36.7X5A | ADVERSE EFFECT OF ANTIFUNGAL ANTIBIOTICS, SYSTEMICALLY USED, INITIAL ENCOUNTER |
|  | T36.7X5D | ADVERSE EFFECT OF ANTIFUNGAL ANTIBIOTICS, SYSTEMICALLY USED, SUBSEQUENT ENCOUNTER |
|  | T36.7X5S | ADVERSE EFFECT OF ANTIFUNGAL ANTIBIOTICS, SYSTEMICALLY USED, SEQUELA |
|  | T36.7X6A | UNDERDOSING OF ANTIFUNGAL ANTIBIOTICS, SYSTEMICALLY USED, INITIAL ENCOUNTER |
|  | T36.7X6D | UNDERDOSING OF ANTIFUNGAL ANTIBIOTICS, SYSTEMICALLY USED, SUBSEQUENT ENCOUNTER |
|  | T36.7X6S | UNDERDOSING OF ANTIFUNGAL ANTIBIOTICS, SYSTEMICALLY USED, SEQUELA |
|  | T36.8X1A | POISONING BY OTHER SYSTEMIC ANTIBIOTICS, ACCIDENTAL (UNINTENTIONAL), INITIAL ENCOUNTER |
|  | T36.8X1D | POISONING BY OTHER SYSTEMIC ANTIBIOTICS, ACCIDENTAL (UNINTENTIONAL), SUBSEQUENT ENCOUNTER |
|  | T36.8X1S | POISONING BY OTHER SYSTEMIC ANTIBIOTICS, ACCIDENTAL (UNINTENTIONAL), SEQUELA |
|  | T36.8X2A | POISONING BY OTHER SYSTEMIC ANTIBIOTICS, INTENTIONAL SELF-HARM, INITIAL ENCOUNTER |
|  | T36.8X2D | POISONING BY OTHER SYSTEMIC ANTIBIOTICS, INTENTIONAL SELF-HARM, SUBSEQUENT ENCOUNTER |
|  | T36.8X2S | POISONING BY OTHER SYSTEMIC ANTIBIOTICS, INTENTIONAL SELF-HARM, SEQUELA |
|  | T36.8X3A | POISONING BY OTHER SYSTEMIC ANTIBIOTICS, ASSAULT, INITIAL ENCOUNTER |
|  | T36.8X3D | POISONING BY OTHER SYSTEMIC ANTIBIOTICS, ASSAULT, SUBSEQUENT ENCOUNTER |
|  | T36.8X3S | POISONING BY OTHER SYSTEMIC ANTIBIOTICS, ASSAULT, SEQUELA |
|  | T36.8X4A | POISONING BY OTHER SYSTEMIC ANTIBIOTICS, UNDETERMINED, INITIAL ENCOUNTER |
|  | T36.8X4D | POISONING BY OTHER SYSTEMIC ANTIBIOTICS, UNDETERMINED, SUBSEQUENT ENCOUNTER |
|  | T36.8X4S | POISONING BY OTHER SYSTEMIC ANTIBIOTICS, UNDETERMINED, SEQUELA |
|  | T36.8X5A | ADVERSE EFFECT OF OTHER SYSTEMIC ANTIBIOTICS, INITIAL ENCOUNTER |
|  | T36.8X5D | ADVERSE EFFECT OF OTHER SYSTEMIC ANTIBIOTICS, SUBSEQUENT ENCOUNTER |
|  | T36.8X5S | ADVERSE EFFECT OF OTHER SYSTEMIC ANTIBIOTICS, SEQUELA |
|  | T36.8X6A | UNDERDOSING OF OTHER SYSTEMIC ANTIBIOTICS, INITIAL ENCOUNTER |
|  | T36.8X6D | UNDERDOSING OF OTHER SYSTEMIC ANTIBIOTICS, SUBSEQUENT ENCOUNTER |
|  | T36.8X6S | UNDERDOSING OF OTHER SYSTEMIC ANTIBIOTICS, SEQUELA |
|  | T36.91XA | POISONING BY UNSPECIFIED SYSTEMIC ANTIBIOTIC, ACCIDENTAL (UNINTENTIONAL), INITIAL ENCOUNTER |
|  | T36.91XD | POISONING BY UNSPECIFIED SYSTEMIC ANTIBIOTIC, ACCIDENTAL (UNINTENTIONAL), SUBSEQUENT ENCOUNTER |
|  | T36.91XS | POISONING BY UNSPECIFIED SYSTEMIC ANTIBIOTIC, ACCIDENTAL (UNINTENTIONAL), SEQUELA |
|  | T36.92XA | POISONING BY UNSPECIFIED SYSTEMIC ANTIBIOTIC, INTENTIONAL SELF-HARM, INITIAL ENCOUNTER |
|  | T36.92XD | POISONING BY UNSPECIFIED SYSTEMIC ANTIBIOTIC, INTENTIONAL SELF-HARM, SUBSEQUENT ENCOUNTER |
|  | T36.92XS | POISONING BY UNSPECIFIED SYSTEMIC ANTIBIOTIC, INTENTIONAL SELF-HARM, SEQUELA |
|  | T36.93XA | POISONING BY UNSPECIFIED SYSTEMIC ANTIBIOTIC, ASSAULT, INITIAL ENCOUNTER |
|  | T36.93XD | POISONING BY UNSPECIFIED SYSTEMIC ANTIBIOTIC, ASSAULT, SUBSEQUENT ENCOUNTER |
|  | T36.93XS | POISONING BY UNSPECIFIED SYSTEMIC ANTIBIOTIC, ASSAULT, SEQUELA |
|  | T36.94XA | POISONING BY UNSPECIFIED SYSTEMIC ANTIBIOTIC, UNDETERMINED, INITIAL ENCOUNTER |
|  | T36.94XD | POISONING BY UNSPECIFIED SYSTEMIC ANTIBIOTIC, UNDETERMINED, SUBSEQUENT ENCOUNTER |
|  | T36.94XS | POISONING BY UNSPECIFIED SYSTEMIC ANTIBIOTIC, UNDETERMINED, SEQUELA |
|  | T36.95XA | ADVERSE EFFECT OF UNSPECIFIED SYSTEMIC ANTIBIOTIC, INITIAL ENCOUNTER |
|  | T36.95XD | ADVERSE EFFECT OF UNSPECIFIED SYSTEMIC ANTIBIOTIC, SUBSEQUENT ENCOUNTER |
|  | T36.95XS | ADVERSE EFFECT OF UNSPECIFIED SYSTEMIC ANTIBIOTIC, SEQUELA |
|  | T36.96XA | UNDERDOSING OF UNSPECIFIED SYSTEMIC ANTIBIOTIC, INITIAL ENCOUNTER |
|  | T36.96XD | UNDERDOSING OF UNSPECIFIED SYSTEMIC ANTIBIOTIC, SUBSEQUENT ENCOUNTER |
|  | T36.96XS | UNDERDOSING OF UNSPECIFIED SYSTEMIC ANTIBIOTIC, SEQUELA |

ICD-9 or 10= International Classification of Diseases, Ninth and Tenth Edition, Clinical Modification
